# Supplementary material for: Pepper power: short-term impact of pepper consumption on the gut bacteriome composition in healthy volunteers
Source: PeerJ. 2024 Dec 13;12:e18707. doi: 10.7717/peerj.18707 (PMC11648697; doi:10.7717/peerj.18707)
Supplement: Supplemental Information 1 [file peerj-12-18707-s001.docx]

| Genus | group1 | group2 | AbundanceGroup1 | AbundanceGroup2 | p | p.adj |
| --- | --- | --- | --- | --- | --- | --- |
| Acinetobacter | Initial | 4_days-pepper | 0.00150488071467299 | 0.000117233294255569 | 0.4 | 1 |
| Acinetobacter | Initial | 4_days-pepperless | 0.00150488071467299 | 0.00129897466117298 | 0.556 | 1 |
| Acinetobacter | Initial | 4_days-normal | 0.00150488071467299 | 0.0023569328718764 | 0.61 | 1 |
| Acinetobacter | Initial | 10_days-normal | 0.00150488071467299 | 0.000333840021959918 | 0.057 | 1 |
| Acinetobacter | 4_days-pepper | 4_days-pepperless | 0.000117233294255569 | 0.00129897466117298 | 0.667 | 1 |
| Acinetobacter | 4_days-pepper | 4_days-normal | 0.000117233294255569 | 0.0023569328718764 | 0.571 | 1 |
| Acinetobacter | 4_days-pepper | 10_days-normal | 0.000117233294255569 | 0.000333840021959918 | 1 | 1 |
| Acinetobacter | 4_days-pepperless | 4_days-normal | 0.00129897466117298 | 0.0023569328718764 | 0.329 | 1 |
| Acinetobacter | 4_days-pepperless | 10_days-normal | 0.00129897466117298 | 0.000333840021959918 | 0.25 | 1 |
| Acinetobacter | 4_days-normal | 10_days-normal | 0.0023569328718764 | 0.000333840021959918 | 0.167 | 1 |
| Akkermansia | Initial | 4_days-pepper | 0.02661818643418 | 0.0749240387683102 | 0.863 | 1 |
| Akkermansia | Initial | 4_days-pepperless | 0.02661818643418 | 0.0253597806781114 | 0.863 | 1 |
| Akkermansia | Initial | 4_days-normal | 0.02661818643418 | 0.0196920562811752 | 0.73 | 1 |
| Akkermansia | Initial | 10_days-normal | 0.02661818643418 | 0.00982981921005845 | 0.021 | 1 |
| Akkermansia | 4_days-pepper | 4_days-pepperless | 0.0749240387683102 | 0.0253597806781114 | 0.546 | 1 |
| Akkermansia | 4_days-pepper | 4_days-normal | 0.0749240387683102 | 0.0196920562811752 | 0.436 | 1 |
| Akkermansia | 4_days-pepper | 10_days-normal | 0.0749240387683102 | 0.00982981921005845 | 0.036 | 1 |
| Akkermansia | 4_days-pepperless | 4_days-normal | 0.0253597806781114 | 0.0196920562811752 | 0.666 | 1 |
| Akkermansia | 4_days-pepperless | 10_days-normal | 0.0253597806781114 | 0.00982981921005845 | 0.027 | 1 |
| Akkermansia | 4_days-normal | 10_days-normal | 0.0196920562811752 | 0.00982981921005845 | 0.059 | 1 |
| Alistipes | 4_days-pepper | 4_days-normal | 0.00072293864790934 | 0.000475484725277486 | 1 | 1 |
| Alistipes | 4_days-pepper | 10_days-normal | 0.00072293864790934 | 0.00886744321988391 | 1 | 1 |
| Alistipes | 4_days-normal | 10_days-normal | 0.000475484725277486 | 0.00886744321988391 | 1 | 1 |
| Allobaculum | Initial | 4_days-pepper | 0.00241322300019493 | 0.00332395522487477 | 1 | 1 |
| Allobaculum | Initial | 4_days-pepperless | 0.00241322300019493 | 0.0202444550626475 | 0.114 | 1 |
| Allobaculum | Initial | 4_days-normal | 0.00241322300019493 | 0.0064291450732347 | 0.683 | 1 |
| Allobaculum | Initial | 10_days-normal | 0.00241322300019493 | 0.00192543909643954 | 0.164 | 1 |
| Allobaculum | 4_days-pepper | 4_days-pepperless | 0.00332395522487477 | 0.0202444550626475 | 0.643 | 1 |
| Allobaculum | 4_days-pepper | 4_days-normal | 0.00332395522487477 | 0.0064291450732347 | 0.889 | 1 |
| Allobaculum | 4_days-pepper | 10_days-normal | 0.00332395522487477 | 0.00192543909643954 | 0.5 | 1 |
| Allobaculum | 4_days-pepperless | 4_days-normal | 0.0202444550626475 | 0.0064291450732347 | 0.108 | 1 |
| Allobaculum | 4_days-pepperless | 10_days-normal | 0.0202444550626475 | 0.00192543909643954 | 0.008 | 1 |
| Allobaculum | 4_days-normal | 10_days-normal | 0.0064291450732347 | 0.00192543909643954 | 0.094 | 1 |
| Anaeroplasma | Initial | 4_days-pepper | 0.00160880690592833 | 0.000476297533485132 | 0.8 | 1 |
| Anaeroplasma | Initial | 4_days-pepperless | 0.00160880690592833 | 0.00159749557008025 | 0.914 | 1 |
| Anaeroplasma | Initial | 4_days-normal | 0.00160880690592833 | 0.00116899091088666 | 1 | 1 |
| Anaeroplasma | Initial | 10_days-normal | 0.00160880690592833 | 0.000838561218660111 | 0.257 | 1 |
| Anaeroplasma | 4_days-pepper | 4_days-pepperless | 0.000476297533485132 | 0.00159749557008025 | 0.857 | 1 |
| Anaeroplasma | 4_days-pepper | 4_days-normal | 0.000476297533485132 | 0.00116899091088666 | 1 | 1 |
| Anaeroplasma | 4_days-pepper | 10_days-normal | 0.000476297533485132 | 0.000838561218660111 | 0.429 | 1 |
| Anaeroplasma | 4_days-pepperless | 4_days-normal | 0.00159749557008025 | 0.00116899091088666 | 0.931 | 1 |
| Anaeroplasma | 4_days-pepperless | 10_days-normal | 0.00159749557008025 | 0.000838561218660111 | 0.093 | 1 |
| Anaeroplasma | 4_days-normal | 10_days-normal | 0.00116899091088666 | 0.000838561218660111 | 0.247 | 1 |
| Arsenophonus | 4_days-normal | 10_days-normal | 0.000236384987265712 | 0.000688261006091722 | 1 | 1 |
| Bacteroides | Initial | 4_days-pepper | 0.210556919114377 | 0.344654729539409 | 0.043 | 1 |
| Bacteroides | Initial | 4_days-pepperless | 0.210556919114377 | 0.273754948901388 | 0.684 | 1 |
| Bacteroides | Initial | 4_days-normal | 0.210556919114377 | 0.161134431295779 | 0.247 | 1 |
| Bacteroides | Initial | 10_days-normal | 0.210556919114377 | 0.364509668001632 | 0.28 | 1 |
| Bacteroides | 4_days-pepper | 4_days-pepperless | 0.344654729539409 | 0.273754948901388 | 0.105 | 1 |
| Bacteroides | 4_days-pepper | 4_days-normal | 0.344654729539409 | 0.161134431295779 | 0.012 | 1 |
| Bacteroides | 4_days-pepper | 10_days-normal | 0.344654729539409 | 0.364509668001632 | 0.684 | 1 |
| Bacteroides | 4_days-pepperless | 4_days-normal | 0.273754948901388 | 0.161134431295779 | 0.631 | 1 |
| Bacteroides | 4_days-pepperless | 10_days-normal | 0.273754948901388 | 0.364509668001632 | 0.353 | 1 |
| Bacteroides | 4_days-normal | 10_days-normal | 0.161134431295779 | 0.364509668001632 | 0.143 | 1 |
| Bifidobacterium | Initial | 4_days-pepper | 0.00296507479744845 | 0.00206107296376933 | 1 | 1 |
| Bifidobacterium | Initial | 4_days-pepperless | 0.00296507479744845 | 0.00532697471412573 | 0.432 | 1 |
| Bifidobacterium | Initial | 4_days-normal | 0.00296507479744845 | 0.00151924439922821 | 0.052 | 1 |
| Bifidobacterium | Initial | 10_days-normal | 0.00296507479744845 | 0.000699864135077323 | 0.016 | 1 |
| Bifidobacterium | 4_days-pepper | 4_days-pepperless | 0.00206107296376933 | 0.00532697471412573 | 1 | 1 |
| Bifidobacterium | 4_days-pepper | 4_days-normal | 0.00206107296376933 | 0.00151924439922821 | 0.857 | 1 |
| Bifidobacterium | 4_days-pepper | 10_days-normal | 0.00206107296376933 | 0.000699864135077323 | 0.533 | 1 |
| Bifidobacterium | 4_days-pepperless | 4_days-normal | 0.00532697471412573 | 0.00151924439922821 | 0.005 | 1 |
| Bifidobacterium | 4_days-pepperless | 10_days-normal | 0.00532697471412573 | 0.000699864135077323 | 0.006 | 1 |
| Bifidobacterium | 4_days-normal | 10_days-normal | 0.00151924439922821 | 0.000699864135077323 | 0.352 | 1 |
| Bilophila | Initial | 4_days-pepper | 4,85E+09 | 0.000266335227272727 | 1 | 1 |
| Bilophila | Initial | 4_days-pepperless | 4,85E+09 | 0.00261193862216185 | 0.5 | 1 |
| Bilophila | Initial | 4_days-normal | 4,85E+09 | 8,39E+09 | 1 | 1 |
| Bilophila | Initial | 10_days-normal | 4,85E+09 | 3,89E+09 | 1 | 1 |
| Bilophila | 4_days-pepper | 4_days-pepperless | 0.000266335227272727 | 0.00261193862216185 | 1 | 1 |
| Bilophila | 4_days-pepper | 4_days-normal | 0.000266335227272727 | 8,39E+09 | 1 | 1 |
| Bilophila | 4_days-pepper | 10_days-normal | 0.000266335227272727 | 3,89E+09 | 1 | 1 |
| Bilophila | 4_days-pepperless | 4_days-normal | 0.00261193862216185 | 8,39E+09 | 1 | 1 |
| Bilophila | 4_days-pepperless | 10_days-normal | 0.00261193862216185 | 3,89E+09 | 0.5 | 1 |
| Bilophila | 4_days-normal | 10_days-normal | 8,39E+09 | 3,89E+09 | 1 | 1 |
| Blautia | Initial | 4_days-pepper | 0.00141212280396894 | 0.000905539772727273 | 0.4 | 1 |
| Blautia | Initial | 4_days-pepperless | 0.00141212280396894 | 0.00161916298329392 | 0.905 | 1 |
| Blautia | Initial | 4_days-normal | 0.00141212280396894 | 0.0017166042367005 | 0.413 | 1 |
| Blautia | Initial | 10_days-normal | 0.00141212280396894 | 0.000818707355083525 | 0.343 | 1 |
| Blautia | 4_days-pepper | 4_days-pepperless | 0.000905539772727273 | 0.00161916298329392 | 0.333 | 1 |
| Blautia | 4_days-pepper | 4_days-normal | 0.000905539772727273 | 0.0017166042367005 | 0.333 | 1 |
| Blautia | 4_days-pepper | 10_days-normal | 0.000905539772727273 | 0.000818707355083525 | 0.4 | 1 |
| Blautia | 4_days-pepperless | 4_days-normal | 0.00161916298329392 | 0.0017166042367005 | 0.31 | 1 |
| Blautia | 4_days-pepperless | 10_days-normal | 0.00161916298329392 | 0.000818707355083525 | 0.413 | 1 |
| Blautia | 4_days-normal | 10_days-normal | 0.0017166042367005 | 0.000818707355083525 | 0.413 | 1 |
| Campylobacter | Initial | 4_days-pepper | 0.00455964679675365 | 0.00141614093407272 | 0.229 | 1 |
| Campylobacter | Initial | 4_days-pepperless | 0.00455964679675365 | 0.00649309616712561 | 0.914 | 1 |
| Campylobacter | Initial | 4_days-normal | 0.00455964679675365 | 0.00448512605619141 | 0.257 | 1 |
| Campylobacter | Initial | 10_days-normal | 0.00455964679675365 | 0.00303535936722318 | 0.067 | 1 |
| Campylobacter | 4_days-pepper | 4_days-pepperless | 0.00141614093407272 | 0.00649309616712561 | 0.095 | 1 |
| Campylobacter | 4_days-pepper | 4_days-normal | 0.00141614093407272 | 0.00448512605619141 | 0.262 | 1 |
| Campylobacter | 4_days-pepper | 10_days-normal | 0.00141614093407272 | 0.00303535936722318 | 0.905 | 1 |
| Campylobacter | 4_days-pepperless | 4_days-normal | 0.00649309616712561 | 0.00448512605619141 | 0.31 | 1 |
| Campylobacter | 4_days-pepperless | 10_days-normal | 0.00649309616712561 | 0.00303535936722318 | 0.041 | 1 |
| Campylobacter | 4_days-normal | 10_days-normal | 0.00448512605619141 | 0.00303535936722318 | 0.18 | 1 |
| Candidatus Arthromitus | Initial | 4_days-pepper | 0.00335062538609918 | 0.000234466588511137 | 0.4 | 1 |
| Candidatus Arthromitus | Initial | 4_days-pepperless | 0.00335062538609918 | 0.0033276118048524 | 0.61 | 1 |
| Candidatus Arthromitus | Initial | 4_days-normal | 0.00335062538609918 | 0.00328102623500942 | 0.914 | 1 |
| Candidatus Arthromitus | Initial | 10_days-normal | 0.00335062538609918 | 0.00258643668239986 | 0.412 | 1 |
| Candidatus Arthromitus | 4_days-pepper | 4_days-pepperless | 0.000234466588511137 | 0.0033276118048524 | 0.571 | 1 |
| Candidatus Arthromitus | 4_days-pepper | 4_days-normal | 0.000234466588511137 | 0.00328102623500942 | 0.286 | 1 |
| Candidatus Arthromitus | 4_days-pepper | 10_days-normal | 0.000234466588511137 | 0.00258643668239986 | 1 | 1 |
| Candidatus Arthromitus | 4_days-pepperless | 4_days-normal | 0.0033276118048524 | 0.00328102623500942 | 0.937 | 1 |
| Candidatus Arthromitus | 4_days-pepperless | 10_days-normal | 0.0033276118048524 | 0.00258643668239986 | 0.295 | 1 |
| Candidatus Arthromitus | 4_days-normal | 10_days-normal | 0.00328102623500942 | 0.00258643668239986 | 0.234 | 1 |
| Candidatus Regiella | Initial | 4_days-pepper | 0.00132552042030366 | 0.000136772176631497 | 0.4 | 1 |
| Candidatus Regiella | Initial | 4_days-pepperless | 0.00132552042030366 | 0.00142154340684441 | 0.556 | 1 |
| Candidatus Regiella | Initial | 4_days-normal | 0.00132552042030366 | 0.00104833933779844 | 0.686 | 1 |
| Candidatus Regiella | Initial | 10_days-normal | 0.00132552042030366 | 0.00125458090645782 | 1 | 1 |
| Candidatus Regiella | 4_days-pepper | 4_days-pepperless | 0.000136772176631497 | 0.00142154340684441 | 0.667 | 1 |
| Candidatus Regiella | 4_days-pepper | 4_days-normal | 0.000136772176631497 | 0.00104833933779844 | 0.8 | 1 |
| Candidatus Regiella | 4_days-pepper | 10_days-normal | 0.000136772176631497 | 0.00125458090645782 | 0.8 | 1 |
| Candidatus Regiella | 4_days-pepperless | 4_days-normal | 0.00142154340684441 | 0.00104833933779844 | 0.905 | 1 |
| Candidatus Regiella | 4_days-pepperless | 10_days-normal | 0.00142154340684441 | 0.00125458090645782 | 0.905 | 1 |
| Candidatus Regiella | 4_days-normal | 10_days-normal | 0.00104833933779844 | 0.00125458090645782 | 0.886 | 1 |
| Candidatus Rhabdochlamydia | Initial | 4_days-pepperless | 0.000643142637974888 | 0.00239836570525252 | 0.571 | 1 |
| Candidatus Rhabdochlamydia | Initial | 4_days-normal | 0.000643142637974888 | 0.00169019358544686 | 1 | 1 |
| Candidatus Rhabdochlamydia | Initial | 10_days-normal | 0.000643142637974888 | 0.000717671916756978 | 0.381 | 1 |
| Candidatus Rhabdochlamydia | 4_days-pepperless | 4_days-normal | 0.00239836570525252 | 0.00169019358544686 | 0.177 | 1 |
| Candidatus Rhabdochlamydia | 4_days-pepperless | 10_days-normal | 0.00239836570525252 | 0.000717671916756978 | 0.008 | 1 |
| Candidatus Rhabdochlamydia | 4_days-normal | 10_days-normal | 0.00169019358544686 | 0.000717671916756978 | 0.126 | 1 |
| Clostridium | Initial | 4_days-pepper | 0.00208975515887195 | 0.00718182834838064 | 0.503 | 1 |
| Clostridium | Initial | 4_days-pepperless | 0.00208975515887195 | 0.00200884261669365 | 0.036 | 1 |
| Clostridium | Initial | 4_days-normal | 0.00208975515887195 | 0.029344058541458 | 0.339 | 1 |
| Clostridium | Initial | 10_days-normal | 0.00208975515887195 | 0.00906796489469776 | 0.172 | 1 |
| Clostridium | 4_days-pepper | 4_days-pepperless | 0.00718182834838064 | 0.00200884261669365 | 0.041 | 1 |
| Clostridium | 4_days-pepper | 4_days-normal | 0.00718182834838064 | 0.029344058541458 | 0.264 | 1 |
| Clostridium | 4_days-pepper | 10_days-normal | 0.00718182834838064 | 0.00906796489469776 | 0.112 | 1 |
| Clostridium | 4_days-pepperless | 4_days-normal | 0.00200884261669365 | 0.029344058541458 | 0.32 | 1 |
| Clostridium | 4_days-pepperless | 10_days-normal | 0.00200884261669365 | 0.00906796489469776 | 0.91 | 1 |
| Clostridium | 4_days-normal | 10_days-normal | 0.029344058541458 | 0.00906796489469776 | 0.3 | 1 |
| Coprococcus | 4_days-pepper | 4_days-pepperless | 0.000230823863636364 | 0.00203045409489642 | 0.5 | 1 |
| Coprococcus | 4_days-pepper | 4_days-normal | 0.000230823863636364 | 0.000488975624219939 | 0.4 | 1 |
| Coprococcus | 4_days-pepper | 10_days-normal | 0.000230823863636364 | 0.00433344388196743 | 1 | 1 |
| Coprococcus | 4_days-pepperless | 4_days-normal | 0.00203045409489642 | 0.000488975624219939 | 0.057 | 1 |
| Coprococcus | 4_days-pepperless | 10_days-normal | 0.00203045409489642 | 0.00433344388196743 | 1 | 1 |
| Coprococcus | 4_days-normal | 10_days-normal | 0.000488975624219939 | 0.00433344388196743 | 0.8 | 1 |
| Corynebacterium | Initial | 4_days-pepper | 0.306220831736975 | 0.177062530515661 | 0.017 | 1 |
| Corynebacterium | Initial | 4_days-pepperless | 0.306220831736975 | 0.294937810401043 | 0.905 | 1 |
| Corynebacterium | Initial | 4_days-normal | 0.306220831736975 | 0.264408815099226 | 0.604 | 1 |
| Corynebacterium | Initial | 10_days-normal | 0.306220831736975 | 0.19451897258732 | 0.094 | 1 |
| Corynebacterium | 4_days-pepper | 4_days-pepperless | 0.177062530515661 | 0.294937810401043 | 0.105 | 1 |
| Corynebacterium | 4_days-pepper | 4_days-normal | 0.177062530515661 | 0.264408815099226 | 0.123 | 1 |
| Corynebacterium | 4_days-pepper | 10_days-normal | 0.177062530515661 | 0.19451897258732 | 0.842 | 1 |
| Corynebacterium | 4_days-pepperless | 4_days-normal | 0.294937810401043 | 0.264408815099226 | 0.631 | 1 |
| Corynebacterium | 4_days-pepperless | 10_days-normal | 0.294937810401043 | 0.19451897258732 | 0.356 | 1 |
| Corynebacterium | 4_days-normal | 10_days-normal | 0.264408815099226 | 0.19451897258732 | 0.278 | 1 |
| Dehalobacterium | Initial | 4_days-pepperless | 0.000152334430983713 | 0.000716970680172516 | 0.333 | 1 |
| Dehalobacterium | Initial | 10_days-normal | 0.000152334430983713 | 2,59E+09 | 0.667 | 1 |
| Dehalobacterium | 4_days-pepperless | 10_days-normal | 0.000716970680172516 | 2,59E+09 | 0.667 | 1 |
| Desulfovibrio | Initial | 4_days-pepper | 0.000279637572856038 | 0.000465791621699468 | 0.8 | 1 |
| Desulfovibrio | Initial | 4_days-pepperless | 0.000279637572856038 | 0.00119565336727083 | 0.429 | 1 |
| Desulfovibrio | Initial | 4_days-normal | 0.000279637572856038 | 0.000819970775283152 | 0.571 | 1 |
| Desulfovibrio | Initial | 10_days-normal | 0.000279637572856038 | 0.000272630034242062 | 1 | 1 |
| Desulfovibrio | 4_days-pepper | 4_days-pepperless | 0.000465791621699468 | 0.00119565336727083 | 0.548 | 1 |
| Desulfovibrio | 4_days-pepper | 4_days-normal | 0.000465791621699468 | 0.000819970775283152 | 0.571 | 1 |
| Desulfovibrio | 4_days-pepper | 10_days-normal | 0.000465791621699468 | 0.000272630034242062 | 0.8 | 1 |
| Desulfovibrio | 4_days-pepperless | 4_days-normal | 0.00119565336727083 | 0.000819970775283152 | 1 | 1 |
| Desulfovibrio | 4_days-pepperless | 10_days-normal | 0.00119565336727083 | 0.000272630034242062 | 0.643 | 1 |
| Desulfovibrio | 4_days-normal | 10_days-normal | 0.000819970775283152 | 0.000272630034242062 | 0.571 | 1 |
| Enhydrobacter | Initial | 4_days-pepper | 0.000785511851187823 | 7,82E+09 | 0.5 | 1 |
| Enhydrobacter | Initial | 4_days-pepperless | 0.000785511851187823 | 0.000705230710453127 | 0.4 | 1 |
| Enhydrobacter | Initial | 4_days-normal | 0.000785511851187823 | 0.000283071145559395 | 0.4 | 1 |
| Enhydrobacter | Initial | 10_days-normal | 0.000785511851187823 | 3,03E+08 | 0.5 | 1 |
| Enhydrobacter | 4_days-pepper | 4_days-pepperless | 7,82E+09 | 0.000705230710453127 | 0.4 | 1 |
| Enhydrobacter | 4_days-pepper | 4_days-normal | 7,82E+09 | 0.000283071145559395 | 1 | 1 |
| Enhydrobacter | 4_days-pepper | 10_days-normal | 7,82E+09 | 3,03E+08 | 1 | 1 |
| Enhydrobacter | 4_days-pepperless | 4_days-normal | 0.000705230710453127 | 0.000283071145559395 | 0.4 | 1 |
| Enhydrobacter | 4_days-pepperless | 10_days-normal | 0.000705230710453127 | 3,03E+08 | 0.4 | 1 |
| Enhydrobacter | 4_days-normal | 10_days-normal | 0.000283071145559395 | 3,03E+08 | 0.5 | 1 |
| Enterococcus | Initial | 4_days-pepper | 0.00137011879736402 | 0.000332161000390778 | 0.667 | 1 |
| Enterococcus | Initial | 4_days-pepperless | 0.00137011879736402 | 0.00218531472124009 | 1 | 1 |
| Enterococcus | Initial | 4_days-normal | 0.00137011879736402 | 0.00190462194891734 | 0.133 | 1 |
| Enterococcus | Initial | 10_days-normal | 0.00137011879736402 | 0.000643365481452403 | 0.333 | 1 |
| Enterococcus | 4_days-pepper | 4_days-pepperless | 0.000332161000390778 | 0.00218531472124009 | 0.5 | 1 |
| Enterococcus | 4_days-pepper | 4_days-normal | 0.000332161000390778 | 0.00190462194891734 | 0.8 | 1 |
| Enterococcus | 4_days-pepper | 10_days-normal | 0.000332161000390778 | 0.000643365481452403 | 1 | 1 |
| Enterococcus | 4_days-pepperless | 4_days-normal | 0.00218531472124009 | 0.00190462194891734 | 0.4 | 1 |
| Enterococcus | 4_days-pepperless | 10_days-normal | 0.00218531472124009 | 0.000643365481452403 | 0.2 | 1 |
| Enterococcus | 4_days-normal | 10_days-normal | 0.00190462194891734 | 0.000643365481452403 | 0.267 | 1 |
| Helicobacter | Initial | 4_days-pepper | 0.00150938217497544 | 0.0025008753689747 | 0.73 | 1 |
| Helicobacter | Initial | 4_days-pepperless | 0.00150938217497544 | 0.00141701507191306 | 0.886 | 1 |
| Helicobacter | Initial | 4_days-normal | 0.00150938217497544 | 0.00110278889168143 | 0.886 | 1 |
| Helicobacter | Initial | 10_days-normal | 0.00150938217497544 | 0.000623534146153427 | 0.064 | 1 |
| Helicobacter | 4_days-pepper | 4_days-pepperless | 0.0025008753689747 | 0.00141701507191306 | 0.73 | 1 |
| Helicobacter | 4_days-pepper | 4_days-normal | 0.0025008753689747 | 0.00110278889168143 | 0.73 | 1 |
| Helicobacter | 4_days-pepper | 10_days-normal | 0.0025008753689747 | 0.000623534146153427 | 0.095 | 1 |
| Helicobacter | 4_days-pepperless | 4_days-normal | 0.00141701507191306 | 0.00110278889168143 | 1 | 1 |
| Helicobacter | 4_days-pepperless | 10_days-normal | 0.00141701507191306 | 0.000623534146153427 | 0.064 | 1 |
| Helicobacter | 4_days-normal | 10_days-normal | 0.00110278889168143 | 0.000623534146153427 | 0.111 | 1 |
| Hespellia | Initial | 4_days-pepper | 0.000598705501618123 | 0.0636181581576318 | 0.667 | 1 |
| Hespellia | Initial | 4_days-normal | 0.000598705501618123 | 0.0029320910375597 | 1 | 1 |
| Hespellia | Initial | 10_days-normal | 0.000598705501618123 | 0.00131944444444444 | 1 | 1 |
| Hespellia | 4_days-pepper | 4_days-normal | 0.0636181581576318 | 0.0029320910375597 | 0.2 | 1 |
| Hespellia | 4_days-pepper | 10_days-normal | 0.0636181581576318 | 0.00131944444444444 | 0.667 | 1 |
| Hespellia | 4_days-normal | 10_days-normal | 0.0029320910375597 | 0.00131944444444444 | 1 | 1 |
| Lactobacillus | Initial | 4_days-pepper | 0.0274730214721762 | 0.0193356338434813 | 0.481 | 1 |
| Lactobacillus | Initial | 4_days-pepperless | 0.0274730214721762 | 0.0357900745919674 | 0.277 | 1 |
| Lactobacillus | Initial | 4_days-normal | 0.0274730214721762 | 0.0505142377499269 | 0.436 | 1 |
| Lactobacillus | Initial | 10_days-normal | 0.0274730214721762 | 0.105009238805037 | 1 | 1 |
| Lactobacillus | 4_days-pepper | 4_days-pepperless | 0.0193356338434813 | 0.0357900745919674 | 0.05 | 1 |
| Lactobacillus | 4_days-pepper | 4_days-normal | 0.0193356338434813 | 0.0505142377499269 | 0.114 | 1 |
| Lactobacillus | 4_days-pepper | 10_days-normal | 0.0193356338434813 | 0.105009238805037 | 0.541 | 1 |
| Lactobacillus | 4_days-pepperless | 4_days-normal | 0.0357900745919674 | 0.0505142377499269 | 0.888 | 1 |
| Lactobacillus | 4_days-pepperless | 10_days-normal | 0.0357900745919674 | 0.105009238805037 | 0.481 | 1 |
| Lactobacillus | 4_days-normal | 10_days-normal | 0.0505142377499269 | 0.105009238805037 | 0.863 | 1 |
| Lactococcus | Initial | 4_days-pepper | 0.0002958188026295 | 9,77E+09 | 0.667 | 1 |
| Lactococcus | Initial | 4_days-pepperless | 0.0002958188026295 | 0.00520544832841026 | 0.133 | 1 |
| Lactococcus | Initial | 4_days-normal | 0.0002958188026295 | 0.00101045735945825 | 1 | 1 |
| Lactococcus | Initial | 10_days-normal | 0.0002958188026295 | 5,10E+09 | 0.333 | 1 |
| Lactococcus | 4_days-pepper | 4_days-pepperless | 9,77E+09 | 0.00520544832841026 | 0.4 | 1 |
| Lactococcus | 4_days-pepper | 4_days-normal | 9,77E+09 | 0.00101045735945825 | 0.4 | 1 |
| Lactococcus | 4_days-pepper | 10_days-normal | 9,77E+09 | 5,10E+09 | 0.667 | 1 |
| Lactococcus | 4_days-pepperless | 4_days-normal | 0.00520544832841026 | 0.00101045735945825 | 0.114 | 1 |
| Lactococcus | 4_days-pepperless | 10_days-normal | 0.00520544832841026 | 5,10E+09 | 0.133 | 1 |
| Lactococcus | 4_days-normal | 10_days-normal | 0.00101045735945825 | 5,10E+09 | 0.133 | 1 |
| Macellibacteroides | Initial | 4_days-pepper | 0.000580564415196415 | 0.00206142696571697 | 0.4 | 1 |
| Macellibacteroides | Initial | 4_days-pepperless | 0.000580564415196415 | 0.000718601992487343 | 0.667 | 1 |
| Macellibacteroides | Initial | 4_days-normal | 0.000580564415196415 | 0.00943703221317414 | 1 | 1 |
| Macellibacteroides | Initial | 10_days-normal | 0.000580564415196415 | 0.0108732389006101 | 0.381 | 1 |
| Macellibacteroides | 4_days-pepper | 4_days-pepperless | 0.00206142696571697 | 0.000718601992487343 | 1 | 1 |
| Macellibacteroides | 4_days-pepper | 4_days-normal | 0.00206142696571697 | 0.00943703221317414 | 0.857 | 1 |
| Macellibacteroides | 4_days-pepper | 10_days-normal | 0.00206142696571697 | 0.0108732389006101 | 1 | 1 |
| Macellibacteroides | 4_days-pepperless | 4_days-normal | 0.000718601992487343 | 0.00943703221317414 | 1 | 1 |
| Macellibacteroides | 4_days-pepperless | 10_days-normal | 0.000718601992487343 | 0.0108732389006101 | 1 | 1 |
| Macellibacteroides | 4_days-normal | 10_days-normal | 0.00943703221317414 | 0.0108732389006101 | 0.556 | 1 |
| Mycoplasma | Initial | 4_days-pepper | 0.19015198044419 | 0.117928783501332 | 0.04 | 1 |
| Mycoplasma | Initial | 4_days-pepperless | 0.19015198044419 | 0.171521922558135 | 0.546 | 1 |
| Mycoplasma | Initial | 4_days-normal | 0.19015198044419 | 0.165930324561567 | 0.605 | 1 |
| Mycoplasma | Initial | 10_days-normal | 0.19015198044419 | 0.107153402398922 | 0.2 | 1 |
| Mycoplasma | 4_days-pepper | 4_days-pepperless | 0.117928783501332 | 0.171521922558135 | 0.136 | 1 |
| Mycoplasma | 4_days-pepper | 4_days-normal | 0.117928783501332 | 0.165930324561567 | 0.05 | 1 |
| Mycoplasma | 4_days-pepper | 10_days-normal | 0.117928783501332 | 0.107153402398922 | 0.963 | 1 |
| Mycoplasma | 4_days-pepperless | 4_days-normal | 0.171521922558135 | 0.165930324561567 | 0.387 | 1 |
| Mycoplasma | 4_days-pepperless | 10_days-normal | 0.171521922558135 | 0.107153402398922 | 0.37 | 1 |
| Mycoplasma | 4_days-normal | 10_days-normal | 0.165930324561567 | 0.107153402398922 | 0.167 | 1 |
| Odoribacter | Initial | 4_days-pepper | 0.00111650485436893 | 0.00641413077507194 | 0.667 | 1 |
| Odoribacter | Initial | 4_days-pepperless | 0.00111650485436893 | 0.000215390640297631 | 1 | 1 |
| Odoribacter | Initial | 4_days-normal | 0.00111650485436893 | 0.0129988534184527 | 1 | 1 |
| Odoribacter | Initial | 10_days-normal | 0.00111650485436893 | 0.00855543627576635 | 1 | 1 |
| Odoribacter | 4_days-pepper | 4_days-pepperless | 0.00641413077507194 | 0.000215390640297631 | 0.667 | 1 |
| Odoribacter | 4_days-pepper | 4_days-normal | 0.00641413077507194 | 0.0129988534184527 | 0.8 | 1 |
| Odoribacter | 4_days-pepper | 10_days-normal | 0.00641413077507194 | 0.00855543627576635 | 0.111 | 1 |
| Odoribacter | 4_days-pepperless | 4_days-normal | 0.000215390640297631 | 0.0129988534184527 | 0.8 | 1 |
| Odoribacter | 4_days-pepperless | 10_days-normal | 0.000215390640297631 | 0.00855543627576635 | 0.5 | 1 |
| Odoribacter | 4_days-normal | 10_days-normal | 0.0129988534184527 | 0.00855543627576635 | 0.788 | 1 |
| Oscillospira | Initial | 4_days-pepper | 0.000654264096285597 | 0.000514914772727273 | 0.5 | 1 |
| Oscillospira | Initial | 4_days-pepperless | 0.000654264096285597 | 0.00680377949430632 | 0.167 | 1 |
| Oscillospira | Initial | 4_days-normal | 0.000654264096285597 | 0.000486540070346599 | 0.229 | 1 |
| Oscillospira | Initial | 10_days-normal | 0.000654264096285597 | 0.000487744721251015 | 0.4 | 1 |
| Oscillospira | 4_days-pepper | 4_days-pepperless | 0.000514914772727273 | 0.00680377949430632 | 1 | 1 |
| Oscillospira | 4_days-pepper | 4_days-normal | 0.000514914772727273 | 0.000486540070346599 | 0.4 | 1 |
| Oscillospira | 4_days-pepper | 10_days-normal | 0.000514914772727273 | 0.000487744721251015 | 0.4 | 1 |
| Oscillospira | 4_days-pepperless | 4_days-normal | 0.00680377949430632 | 0.000486540070346599 | 0.067 | 1 |
| Oscillospira | 4_days-pepperless | 10_days-normal | 0.00680377949430632 | 0.000487744721251015 | 0.067 | 1 |
| Oscillospira | 4_days-normal | 10_days-normal | 0.000486540070346599 | 0.000487744721251015 | 0.886 | 1 |
| Parabacteroides | Initial | 4_days-pepper | 0.00133130528245306 | 0.00632735760967121 | 0.057 | 1 |
| Parabacteroides | Initial | 4_days-pepperless | 0.00133130528245306 | 0.00364935924096551 | 0.25 | 1 |
| Parabacteroides | Initial | 4_days-normal | 0.00133130528245306 | 0.00219523053624672 | 0.667 | 1 |
| Parabacteroides | Initial | 10_days-normal | 0.00133130528245306 | 0.000371331335964717 | 0.2 | 1 |
| Parabacteroides | 4_days-pepper | 4_days-pepperless | 0.00632735760967121 | 0.00364935924096551 | 0.064 | 1 |
| Parabacteroides | 4_days-pepper | 4_days-normal | 0.00632735760967121 | 0.00219523053624672 | 0.006 | 1 |
| Parabacteroides | 4_days-pepper | 10_days-normal | 0.00632735760967121 | 0.000371331335964717 | 0.057 | 1 |
| Parabacteroides | 4_days-pepperless | 4_days-normal | 0.00364935924096551 | 0.00219523053624672 | 0.01 | 1 |
| Parabacteroides | 4_days-pepperless | 10_days-normal | 0.00364935924096551 | 0.000371331335964717 | 0.036 | 1 |
| Parabacteroides | 4_days-normal | 10_days-normal | 0.00219523053624672 | 0.000371331335964717 | 0.067 | 1 |
| Prevotella | 4_days-pepper | 4_days-pepperless | 0.0136844343223941 | 0.00415698632354369 | 0.413 | 1 |
| Prevotella | 4_days-pepper | 4_days-normal | 0.0136844343223941 | 0.000106007208877377 | 0.133 | 1 |
| Prevotella | 4_days-pepper | 10_days-normal | 0.0136844343223941 | 0.000257731958762887 | 0.8 | 1 |
| Prevotella | 4_days-pepperless | 4_days-normal | 0.00415698632354369 | 0.000106007208877377 | 0.095 | 1 |
| Prevotella | 4_days-pepperless | 10_days-normal | 0.00415698632354369 | 0.000257731958762887 | 0.333 | 1 |
| Prevotella | 4_days-normal | 10_days-normal | 0.000106007208877377 | 0.000257731958762887 | 0.667 | 1 |
| Pseudomonas | Initial | 4_days-pepperless | 0.00311319568664375 | 0.00385435300917513 | 0.476 | 1 |
| Pseudomonas | Initial | 4_days-normal | 0.00311319568664375 | 0.00257948772201858 | 0.886 | 1 |
| Pseudomonas | Initial | 10_days-normal | 0.00311319568664375 | 0.00160519221431887 | 0.067 | 1 |
| Pseudomonas | 4_days-pepperless | 4_days-normal | 0.00385435300917513 | 0.00257948772201858 | 0.61 | 1 |
| Pseudomonas | 4_days-pepperless | 10_days-normal | 0.00385435300917513 | 0.00160519221431887 | 0.132 | 1 |
| Pseudomonas | 4_days-normal | 10_days-normal | 0.00257948772201858 | 0.00160519221431887 | 0.067 | 1 |
| Rickettsiella | Initial | 4_days-pepperless | 0.000471698113207547 | 0.000688733729383228 | 0.5 | 1 |
| Rickettsiella | Initial | 10_days-normal | 0.000471698113207547 | 0.000545884335086031 | 0.667 | 1 |
| Rickettsiella | 4_days-pepperless | 10_days-normal | 0.000688733729383228 | 0.000545884335086031 | 1 | 1 |
| Rothia | Initial | 4_days-pepperless | 0.000225281602002503 | 0.00072139323129781 | 1 | 1 |
| Rothia | Initial | 4_days-normal | 0.000225281602002503 | 0.00105648806965772 | 1 | 1 |
| Rothia | Initial | 10_days-normal | 0.000225281602002503 | 0.00024091902006911 | 0.667 | 1 |
| Rothia | 4_days-pepperless | 4_days-normal | 0.00072139323129781 | 0.00105648806965772 | 0.629 | 1 |
| Rothia | 4_days-pepperless | 10_days-normal | 0.00072139323129781 | 0.00024091902006911 | 0.2 | 1 |
| Rothia | 4_days-normal | 10_days-normal | 0.00105648806965772 | 0.00024091902006911 | 0.133 | 1 |
| Ruminococcus | Initial | 4_days-pepper | 0.00053650254121382 | 0.010848403815843 | 0.057 | 1 |
| Ruminococcus | Initial | 4_days-pepperless | 0.00053650254121382 | 0.000524610520784852 | 0.629 | 1 |
| Ruminococcus | Initial | 4_days-normal | 0.00053650254121382 | 0.00460185322720154 | 0.26 | 1 |
| Ruminococcus | Initial | 10_days-normal | 0.00053650254121382 | 0.02697338708972 | 0.77 | 1 |
| Ruminococcus | 4_days-pepper | 4_days-pepperless | 0.010848403815843 | 0.000524610520784852 | 0.1 | 1 |
| Ruminococcus | 4_days-pepper | 4_days-normal | 0.010848403815843 | 0.00460185322720154 | 0.1 | 1 |
| Ruminococcus | 4_days-pepper | 10_days-normal | 0.010848403815843 | 0.02697338708972 | 0.295 | 1 |
| Ruminococcus | 4_days-pepperless | 4_days-normal | 0.000524610520784852 | 0.00460185322720154 | 0.864 | 1 |
| Ruminococcus | 4_days-pepperless | 10_days-normal | 0.000524610520784852 | 0.02697338708972 | 1 | 1 |
| Ruminococcus | 4_days-normal | 10_days-normal | 0.00460185322720154 | 0.02697338708972 | 0.702 | 1 |
| Shigella | Initial | 4_days-pepper | 0.124379444368297 | 0.0602577247555896 | 0.263 | 1 |
| Shigella | Initial | 4_days-pepperless | 0.124379444368297 | 0.0567849500762252 | 0.016 | 1 |
| Shigella | Initial | 4_days-normal | 0.124379444368297 | 0.0718512402317263 | 0.023 | 1 |
| Shigella | Initial | 10_days-normal | 0.124379444368297 | 0.0379172748183179 | 0.001 | 0.408 |
| Shigella | 4_days-pepper | 4_days-pepperless | 0.0602577247555896 | 0.0567849500762252 | 0.282 | 1 |
| Shigella | 4_days-pepper | 4_days-normal | 0.0602577247555896 | 0.0718512402317263 | 0.562 | 1 |
| Shigella | 4_days-pepper | 10_days-normal | 0.0602577247555896 | 0.0379172748183179 | 0.066 | 1 |
| Shigella | 4_days-pepperless | 4_days-normal | 0.0567849500762252 | 0.0718512402317263 | 0.762 | 1 |
| Shigella | 4_days-pepperless | 10_days-normal | 0.0567849500762252 | 0.0379172748183179 | 0.027 | 1 |
| Shigella | 4_days-normal | 10_days-normal | 0.0718512402317263 | 0.0379172748183179 | 0.028 | 1 |
| Staphylococcus | Initial | 4_days-pepper | 0.0661632798215047 | 0.0325431873982194 | 0.021 | 1 |
| Staphylococcus | Initial | 4_days-pepperless | 0.0661632798215047 | 0.0357635539105756 | 0.001 | 0.408 |
| Staphylococcus | Initial | 4_days-normal | 0.0661632798215047 | 0.0431732940927342 | 0.017 | 1 |
| Staphylococcus | Initial | 10_days-normal | 0.0661632798215047 | 0.0169829422400893 | 0.000699 | 0.285891 |
| Staphylococcus | 4_days-pepper | 4_days-pepperless | 0.0325431873982194 | 0.0357635539105756 | 1 | 1 |
| Staphylococcus | 4_days-pepper | 4_days-normal | 0.0325431873982194 | 0.0431732940927342 | 0.897 | 1 |
| Staphylococcus | 4_days-pepper | 10_days-normal | 0.0325431873982194 | 0.0169829422400893 | 0.281 | 1 |
| Staphylococcus | 4_days-pepperless | 4_days-normal | 0.0357635539105756 | 0.0431732940927342 | 0.604 | 1 |
| Staphylococcus | 4_days-pepperless | 10_days-normal | 0.0357635539105756 | 0.0169829422400893 | 0.142 | 1 |
| Staphylococcus | 4_days-normal | 10_days-normal | 0.0431732940927342 | 0.0169829422400893 | 0.109 | 1 |
| Stenotrophomonas | Initial | 4_days-pepperless | 0.000231877462110906 | 0.000120603015075377 | 1 | 1 |
| Stenotrophomonas | Initial | 4_days-normal | 0.000231877462110906 | 8,39E+09 | 0.667 | 1 |
| Stenotrophomonas | Initial | 10_days-normal | 0.000231877462110906 | 7,21E+09 | 0.667 | 1 |
| Stenotrophomonas | 4_days-pepperless | 4_days-normal | 0.000120603015075377 | 8,39E+09 | 1 | 1 |
| Stenotrophomonas | 4_days-pepperless | 10_days-normal | 0.000120603015075377 | 7,21E+09 | 1 | 1 |
| Stenotrophomonas | 4_days-normal | 10_days-normal | 8,39E+09 | 7,21E+09 | 1 | 1 |
| Streptococcus | Initial | 4_days-pepper | 0.00104962133931272 | 0.00149725274725275 | 0.667 | 1 |
| Streptococcus | Initial | 4_days-pepperless | 0.00104962133931272 | 0.000224358974358974 | 1 | 1 |
| Streptococcus | Initial | 4_days-normal | 0.00104962133931272 | 0.000805313759818623 | 0.8 | 1 |
| Streptococcus | Initial | 10_days-normal | 0.00104962133931272 | 0.0103584510717712 | 0.857 | 1 |
| Streptococcus | 4_days-pepper | 4_days-pepperless | 0.00149725274725275 | 0.000224358974358974 | 1 | 1 |
| Streptococcus | 4_days-pepper | 4_days-normal | 0.00149725274725275 | 0.000805313759818623 | 0.4 | 1 |
| Streptococcus | 4_days-pepper | 10_days-normal | 0.00149725274725275 | 0.0103584510717712 | 1 | 1 |
| Streptococcus | 4_days-pepperless | 4_days-normal | 0.000224358974358974 | 0.000805313759818623 | 0.8 | 1 |
| Streptococcus | 4_days-pepperless | 10_days-normal | 0.000224358974358974 | 0.0103584510717712 | 1 | 1 |
| Streptococcus | 4_days-normal | 10_days-normal | 0.000805313759818623 | 0.0103584510717712 | 0.413 | 1 |
| Succinispira | Initial | 4_days-pepper | 0.000963242955538816 | 0.0019015079557091 | 0.4 | 1 |
| Succinispira | Initial | 4_days-normal | 0.000963242955538816 | 0.000213509020756127 | 1 | 1 |
| Succinispira | 4_days-pepper | 4_days-normal | 0.0019015079557091 | 0.000213509020756127 | 0.667 | 1 |
| Sutterella | Initial | 4_days-pepper | 0.00151561872895734 | 0.00630198483863985 | 0.73 | 1 |
| Sutterella | Initial | 4_days-pepperless | 0.00151561872895734 | 0.00441682391398272 | 0.61 | 1 |
| Sutterella | Initial | 4_days-normal | 0.00151561872895734 | 0.00109731350325421 | 0.111 | 1 |
| Sutterella | Initial | 10_days-normal | 0.00151561872895734 | 0.000907247481384833 | 0.064 | 1 |
| Sutterella | 4_days-pepper | 4_days-pepperless | 0.00630198483863985 | 0.00441682391398272 | 1 | 1 |
| Sutterella | 4_days-pepper | 4_days-normal | 0.00630198483863985 | 0.00109731350325421 | 0.421 | 1 |
| Sutterella | 4_days-pepper | 10_days-normal | 0.00630198483863985 | 0.000907247481384833 | 0.421 | 1 |
| Sutterella | 4_days-pepperless | 4_days-normal | 0.00441682391398272 | 0.00109731350325421 | 0.177 | 1 |
| Sutterella | 4_days-pepperless | 10_days-normal | 0.00441682391398272 | 0.000907247481384833 | 0.082 | 1 |
| Sutterella | 4_days-normal | 10_days-normal | 0.00109731350325421 | 0.000907247481384833 | 0.841 | 1 |
| Swaminathania | Initial | 10_days-normal | 8,76E+09 | 0.000206784130004441 | 1 | 1 |
| Thermus | Initial | 4_days-pepperless | 0.000358397025407974 | 0.000803987992377751 | 0.8 | 1 |
| Thermus | Initial | 4_days-normal | 0.000358397025407974 | 0.000835847418100133 | 0.533 | 1 |
| Thermus | Initial | 10_days-normal | 0.000358397025407974 | 0.000943752725023082 | 1 | 1 |
| Thermus | 4_days-pepperless | 4_days-normal | 0.000803987992377751 | 0.000835847418100133 | 0.343 | 1 |
| Thermus | 4_days-pepperless | 10_days-normal | 0.000803987992377751 | 0.000943752725023082 | 0.886 | 1 |
| Thermus | 4_days-normal | 10_days-normal | 0.000835847418100133 | 0.000943752725023082 | 0.686 | 1 |
| Turicibacter | 4_days-pepperless | 4_days-normal | 0.00852612434699666 | 0.00173238416015824 | 0.111 | 1 |
| Turicibacter | 4_days-pepperless | 10_days-normal | 0.00852612434699666 | 1,56E+09 | 0.333 | 1 |
| Turicibacter | 4_days-normal | 10_days-normal | 0.00173238416015824 | 1,56E+09 | 0.4 | 1 |
| Weissella | Initial | 4_days-normal | 3,75E+09 | 0.000129812071844283 | 1 | 1 |
| Weissella | Initial | 10_days-normal | 3,75E+09 | 6,18E+09 | 1 | 1 |
| Weissella | 4_days-normal | 10_days-normal | 0.000129812071844283 | 6,18E+09 | 1 | 1 |
| Williamsia | Initial | 4_days-pepperless | 0.000354731440190205 | 0.000297323181354903 | 1 | 1 |
| Williamsia | Initial | 4_days-normal | 0.000354731440190205 | 0.000580576064134648 | 1 | 1 |
| Williamsia | Initial | 10_days-normal | 0.000354731440190205 | 0.00021150876854993 | 0.4 | 1 |
| Williamsia | 4_days-pepperless | 4_days-normal | 0.000297323181354903 | 0.000580576064134648 | 1 | 1 |
| Williamsia | 4_days-pepperless | 10_days-normal | 0.000297323181354903 | 0.00021150876854993 | 0.8 | 1 |
| Williamsia | 4_days-normal | 10_days-normal | 0.000580576064134648 | 0.00021150876854993 | 0.4 | 1 |
