# Supplementary material for: Pepper power: short-term impact of pepper consumption on the gut bacteriome composition in healthy volunteers
Source: PeerJ. 2024 Dec 13;12:e18707. doi: 10.7717/peerj.18707 (PMC11648697; doi:10.7717/peerj.18707)
Supplement: Supplemental Information 2 [file peerj-12-18707-s002.docx]

| Family | group1 | group2 | AbundanceGroup1 | AbundanceGroup2 | p | p.adj |
| --- | --- | --- | --- | --- | --- | --- |
| Acetobacteraceae | Initial | 4_days-pepper | 0.00012099568231453 | 7,73E+09 | 0.667 | 1 |
| Acetobacteraceae | Initial | 4_days-pepperless | 0.00012099568231453 | 6,57E+09 | 1 | 1 |
| Acetobacteraceae | Initial | 4_days-normal | 0.00012099568231453 | 0.000121868753215573 | 1 | 1 |
| Acetobacteraceae | Initial | 10_days-normal | 0.00012099568231453 | 0.000151888752278798 | 0.667 | 1 |
| Acetobacteraceae | 4_days-pepper | 4_days-pepperless | 7,73E+09 | 6,57E+09 | 0.667 | 1 |
| Acetobacteraceae | 4_days-pepper | 4_days-normal | 7,73E+09 | 0.000121868753215573 | 0.333 | 1 |
| Acetobacteraceae | 4_days-pepper | 10_days-normal | 7,73E+09 | 0.000151888752278798 | 0.667 | 1 |
| Acetobacteraceae | 4_days-pepperless | 4_days-normal | 6,57E+09 | 0.000121868753215573 | 0.667 | 1 |
| Acetobacteraceae | 4_days-pepperless | 10_days-normal | 6,57E+09 | 0.000151888752278798 | 1 | 1 |
| Acetobacteraceae | 4_days-normal | 10_days-normal | 0.000121868753215573 | 0.000151888752278798 | 1 | 1 |
| Alcaligenaceae | Initial | 4_days-pepper | 0.00103950349314264 | 0.00503831929443781 | 0.73 | 1 |
| Alcaligenaceae | Initial | 4_days-pepperless | 0.00103950349314264 | 0.00286139815922192 | 0.762 | 1 |
| Alcaligenaceae | Initial | 4_days-normal | 0.00103950349314264 | 0.000768052785566546 | 0.19 | 1 |
| Alcaligenaceae | Initial | 10_days-normal | 0.00103950349314264 | 0.000692702878054186 | 0.064 | 1 |
| Alcaligenaceae | 4_days-pepper | 4_days-pepperless | 0.00503831929443781 | 0.00286139815922192 | 0.931 | 1 |
| Alcaligenaceae | 4_days-pepper | 4_days-normal | 0.00503831929443781 | 0.000768052785566546 | 0.421 | 1 |
| Alcaligenaceae | 4_days-pepper | 10_days-normal | 0.00503831929443781 | 0.000692702878054186 | 0.421 | 1 |
| Alcaligenaceae | 4_days-pepperless | 4_days-normal | 0.00286139815922192 | 0.000768052785566546 | 0.177 | 1 |
| Alcaligenaceae | 4_days-pepperless | 10_days-normal | 0.00286139815922192 | 0.000692702878054186 | 0.082 | 1 |
| Alcaligenaceae | 4_days-normal | 10_days-normal | 0.000768052785566546 | 0.000692702878054186 | 1 | 1 |
| Anaeroplasmataceae | Initial | 4_days-pepper | 0.00108119117911961 | 0.000348864583552112 | 0.8 | 1 |
| Anaeroplasmataceae | Initial | 4_days-pepperless | 0.00108119117911961 | 0.00106286627348083 | 1 | 1 |
| Anaeroplasmataceae | Initial | 4_days-normal | 0.00108119117911961 | 0.000825104272591911 | 1 | 1 |
| Anaeroplasmataceae | Initial | 10_days-normal | 0.00108119117911961 | 0.000624699165680524 | 0.257 | 1 |
| Anaeroplasmataceae | 4_days-pepper | 4_days-pepperless | 0.000348864583552112 | 0.00106286627348083 | 1 | 1 |
| Anaeroplasmataceae | 4_days-pepper | 4_days-normal | 0.000348864583552112 | 0.000825104272591911 | 1 | 1 |
| Anaeroplasmataceae | 4_days-pepper | 10_days-normal | 0.000348864583552112 | 0.000624699165680524 | 0.429 | 1 |
| Anaeroplasmataceae | 4_days-pepperless | 4_days-normal | 0.00106286627348083 | 0.000825104272591911 | 0.792 | 1 |
| Anaeroplasmataceae | 4_days-pepperless | 10_days-normal | 0.00106286627348083 | 0.000624699165680524 | 0.24 | 1 |
| Anaeroplasmataceae | 4_days-normal | 10_days-normal | 0.000825104272591911 | 0.000624699165680524 | 0.177 | 1 |
| Bacillaceae | 4_days-pepper | 4_days-pepperless | 0.000149950016661113 | 0.000620471656890602 | 1 | 1 |
| Bacillaceae | 4_days-pepper | 4_days-normal | 0.000149950016661113 | 0.000624873603599964 | 0.667 | 1 |
| Bacillaceae | 4_days-pepper | 10_days-normal | 0.000149950016661113 | 0.00037117903930131 | 1 | 1 |
| Bacillaceae | 4_days-pepperless | 4_days-normal | 0.000620471656890602 | 0.000624873603599964 | 1 | 1 |
| Bacillaceae | 4_days-pepperless | 10_days-normal | 0.000620471656890602 | 0.00037117903930131 | 1 | 1 |
| Bacillaceae | 4_days-normal | 10_days-normal | 0.000624873603599964 | 0.00037117903930131 | 1 | 1 |
| Bacteroidaceae | Initial | 4_days-pepper | 0.179433021373373 | 0.292884730528955 | 0.052 | 1 |
| Bacteroidaceae | Initial | 4_days-pepperless | 0.179433021373373 | 0.230385103497795 | 0.684 | 1 |
| Bacteroidaceae | Initial | 4_days-normal | 0.179433021373373 | 0.139013312238584 | 0.28 | 1 |
| Bacteroidaceae | Initial | 10_days-normal | 0.179433021373373 | 0.327492082323851 | 0.247 | 1 |
| Bacteroidaceae | 4_days-pepper | 4_days-pepperless | 0.292884730528955 | 0.230385103497795 | 0.063 | 1 |
| Bacteroidaceae | 4_days-pepper | 4_days-normal | 0.292884730528955 | 0.139013312238584 | 0.015 | 1 |
| Bacteroidaceae | 4_days-pepper | 10_days-normal | 0.292884730528955 | 0.327492082323851 | 0.739 | 1 |
| Bacteroidaceae | 4_days-pepperless | 4_days-normal | 0.230385103497795 | 0.139013312238584 | 1 | 1 |
| Bacteroidaceae | 4_days-pepperless | 10_days-normal | 0.230385103497795 | 0.327492082323851 | 0.19 | 1 |
| Bacteroidaceae | 4_days-normal | 10_days-normal | 0.139013312238584 | 0.327492082323851 | 0.123 | 1 |
| Bartonellaceae | Initial | 4_days-pepper | 0.00571413866829457 | 0.00372853396557762 | 0.762 | 1 |
| Bartonellaceae | Initial | 4_days-pepperless | 0.00571413866829457 | 0.00883338236958361 | 0.662 | 1 |
| Bartonellaceae | Initial | 4_days-normal | 0.00571413866829457 | 0.00837201594376035 | 0.414 | 1 |
| Bartonellaceae | Initial | 10_days-normal | 0.00571413866829457 | 0.00641505745558742 | 0.529 | 1 |
| Bartonellaceae | 4_days-pepper | 4_days-pepperless | 0.00372853396557762 | 0.00883338236958361 | 0.461 | 1 |
| Bartonellaceae | 4_days-pepper | 4_days-normal | 0.00372853396557762 | 0.00837201594376035 | 0.283 | 1 |
| Bartonellaceae | 4_days-pepper | 10_days-normal | 0.00372853396557762 | 0.00641505745558742 | 0.503 | 1 |
| Bartonellaceae | 4_days-pepperless | 4_days-normal | 0.00883338236958361 | 0.00837201594376035 | 0.798 | 1 |
| Bartonellaceae | 4_days-pepperless | 10_days-normal | 0.00883338236958361 | 0.00641505745558742 | 0.139 | 1 |
| Bartonellaceae | 4_days-normal | 10_days-normal | 0.00837201594376035 | 0.00641505745558742 | 0.139 | 1 |
| Beijerinckiaceae | Initial | 4_days-normal | 0.000514935653155382 | 5,62E+09 | 0.8 | 1 |
| Beijerinckiaceae | Initial | 10_days-normal | 0.000514935653155382 | 2,33E+09 | 0.4 | 1 |
| Beijerinckiaceae | 4_days-normal | 10_days-normal | 5,62E+09 | 2,33E+09 | 1 | 1 |
| Bifidobacteriaceae | Initial | 4_days-pepper | 0.00206966660102145 | 0.00164844041885028 | 1 | 1 |
| Bifidobacteriaceae | Initial | 4_days-pepperless | 0.00206966660102145 | 0.00349244693720764 | 0.432 | 1 |
| Bifidobacteriaceae | Initial | 4_days-normal | 0.00206966660102145 | 0.00110064720576408 | 0.126 | 1 |
| Bifidobacteriaceae | Initial | 10_days-normal | 0.00206966660102145 | 0.000533996231582386 | 0.064 | 1 |
| Bifidobacteriaceae | 4_days-pepper | 4_days-pepperless | 0.00164844041885028 | 0.00349244693720764 | 1 | 1 |
| Bifidobacteriaceae | 4_days-pepper | 4_days-normal | 0.00164844041885028 | 0.00110064720576408 | 0.857 | 1 |
| Bifidobacteriaceae | 4_days-pepper | 10_days-normal | 0.00164844041885028 | 0.000533996231582386 | 0.533 | 1 |
| Bifidobacteriaceae | 4_days-pepperless | 4_days-normal | 0.00349244693720764 | 0.00110064720576408 | 0.005 | 1 |
| Bifidobacteriaceae | 4_days-pepperless | 10_days-normal | 0.00349244693720764 | 0.000533996231582386 | 0.006 | 1 |
| Bifidobacteriaceae | 4_days-normal | 10_days-normal | 0.00110064720576408 | 0.000533996231582386 | 0.476 | 1 |
| Campylobacteraceae | Initial | 4_days-pepper | 0.00309822348513471 | 0.00115369141951807 | 0.229 | 1 |
| Campylobacteraceae | Initial | 4_days-pepperless | 0.00309822348513471 | 0.00452797945131641 | 0.914 | 1 |
| Campylobacteraceae | Initial | 4_days-normal | 0.00309822348513471 | 0.00314082710026205 | 0.352 | 1 |
| Campylobacteraceae | Initial | 10_days-normal | 0.00309822348513471 | 0.00229390228205212 | 0.067 | 1 |
| Campylobacteraceae | 4_days-pepper | 4_days-pepperless | 0.00115369141951807 | 0.00452797945131641 | 0.167 | 1 |
| Campylobacteraceae | 4_days-pepper | 4_days-normal | 0.00115369141951807 | 0.00314082710026205 | 0.548 | 1 |
| Campylobacteraceae | 4_days-pepper | 10_days-normal | 0.00115369141951807 | 0.00229390228205212 | 0.905 | 1 |
| Campylobacteraceae | 4_days-pepperless | 4_days-normal | 0.00452797945131641 | 0.00314082710026205 | 0.394 | 1 |
| Campylobacteraceae | 4_days-pepperless | 10_days-normal | 0.00452797945131641 | 0.00229390228205212 | 0.065 | 1 |
| Campylobacteraceae | 4_days-normal | 10_days-normal | 0.00314082710026205 | 0.00229390228205212 | 0.24 | 1 |
| Clostridiaceae | Initial | 4_days-pepper | 0.00261607295770178 | 0.000199933355548151 | 0.4 | 1 |
| Clostridiaceae | Initial | 4_days-pepperless | 0.00261607295770178 | 0.00274586788799091 | 1 | 1 |
| Clostridiaceae | Initial | 4_days-normal | 0.00261607295770178 | 0.00485453368740969 | 0.788 | 1 |
| Clostridiaceae | Initial | 10_days-normal | 0.00261607295770178 | 0.0122209717495477 | 0.94 | 1 |
| Clostridiaceae | 4_days-pepper | 4_days-pepperless | 0.000199933355548151 | 0.00274586788799091 | 0.286 | 1 |
| Clostridiaceae | 4_days-pepper | 4_days-normal | 0.000199933355548151 | 0.00485453368740969 | 0.25 | 1 |
| Clostridiaceae | 4_days-pepper | 10_days-normal | 0.000199933355548151 | 0.0122209717495477 | 0.6 | 1 |
| Clostridiaceae | 4_days-pepperless | 4_days-normal | 0.00274586788799091 | 0.00485453368740969 | 0.534 | 1 |
| Clostridiaceae | 4_days-pepperless | 10_days-normal | 0.00274586788799091 | 0.0122209717495477 | 0.955 | 1 |
| Clostridiaceae | 4_days-normal | 10_days-normal | 0.00485453368740969 | 0.0122209717495477 | 0.758 | 1 |
| Corynebacteriaceae | Initial | 4_days-pepper | 0.222887192551613 | 0.13876824675861 | 0.054 | 1 |
| Corynebacteriaceae | Initial | 4_days-pepperless | 0.222887192551613 | 0.195431591707725 | 0.243 | 1 |
| Corynebacteriaceae | Initial | 4_days-normal | 0.222887192551613 | 0.194801941532309 | 0.842 | 1 |
| Corynebacteriaceae | Initial | 10_days-normal | 0.222887192551613 | 0.146955388185145 | 0.077 | 1 |
| Corynebacteriaceae | 4_days-pepper | 4_days-pepperless | 0.13876824675861 | 0.195431591707725 | 0.315 | 1 |
| Corynebacteriaceae | 4_days-pepper | 4_days-normal | 0.13876824675861 | 0.194801941532309 | 0.143 | 1 |
| Corynebacteriaceae | 4_days-pepper | 10_days-normal | 0.13876824675861 | 0.146955388185145 | 0.72 | 1 |
| Corynebacteriaceae | 4_days-pepperless | 4_days-normal | 0.195431591707725 | 0.194801941532309 | 0.579 | 1 |
| Corynebacteriaceae | 4_days-pepperless | 10_days-normal | 0.195431591707725 | 0.146955388185145 | 0.497 | 1 |
| Corynebacteriaceae | 4_days-normal | 10_days-normal | 0.194801941532309 | 0.146955388185145 | 0.315 | 1 |
| Coxiellaceae | Initial | 4_days-pepperless | 0.000322997416020672 | 0.000472697649133938 | 0.5 | 1 |
| Coxiellaceae | Initial | 10_days-normal | 0.000322997416020672 | 0.000427980082510012 | 0.667 | 1 |
| Coxiellaceae | 4_days-pepperless | 10_days-normal | 0.000472697649133938 | 0.000427980082510012 | 1 | 1 |
| Dehalobacteriaceae | Initial | 4_days-pepperless | 0.000106715751522129 | 0.000469344517576956 | 0.333 | 1 |
| Dehalobacteriaceae | Initial | 10_days-normal | 0.000106715751522129 | 2,33E+09 | 0.667 | 1 |
| Dehalobacteriaceae | 4_days-pepperless | 10_days-normal | 0.000469344517576956 | 2,33E+09 | 0.667 | 1 |
| Desulfovibrionaceae | Initial | 4_days-pepper | 0.000230952148711379 | 0.000547049096516277 | 0.8 | 1 |
| Desulfovibrionaceae | Initial | 4_days-pepperless | 0.000230952148711379 | 0.00242237211483965 | 0.429 | 1 |
| Desulfovibrionaceae | Initial | 4_days-normal | 0.000230952148711379 | 0.000625939666788613 | 0.571 | 1 |
| Desulfovibrionaceae | Initial | 10_days-normal | 0.000230952148711379 | 0.000228613658446557 | 0.4 | 1 |
| Desulfovibrionaceae | 4_days-pepper | 4_days-pepperless | 0.000547049096516277 | 0.00242237211483965 | 0.905 | 1 |
| Desulfovibrionaceae | 4_days-pepper | 4_days-normal | 0.000547049096516277 | 0.000625939666788613 | 0.571 | 1 |
| Desulfovibrionaceae | 4_days-pepper | 10_days-normal | 0.000547049096516277 | 0.000228613658446557 | 0.7 | 1 |
| Desulfovibrionaceae | 4_days-pepperless | 4_days-normal | 0.00242237211483965 | 0.000625939666788613 | 0.429 | 1 |
| Desulfovibrionaceae | 4_days-pepperless | 10_days-normal | 0.00242237211483965 | 0.000228613658446557 | 0.095 | 1 |
| Desulfovibrionaceae | 4_days-normal | 10_days-normal | 0.000625939666788613 | 0.000228613658446557 | 0.25 | 1 |
| Elusimicrobiaceae | Initial | 4_days-pepper | 0.0019669551534225 | 0.000385420368437107 | 0.667 | 1 |
| Elusimicrobiaceae | Initial | 4_days-normal | 0.0019669551534225 | 0.0581818181818182 | 1 | 1 |
| Elusimicrobiaceae | 4_days-pepper | 4_days-normal | 0.000385420368437107 | 0.0581818181818182 | 0.667 | 1 |
| Enterobacteriaceae | Initial | 4_days-pepper | 0.143159220017511 | 0.0879871554213148 | 0.095 | 1 |
| Enterobacteriaceae | Initial | 4_days-pepperless | 0.143159220017511 | 0.0856580124227717 | 0.054 | 1 |
| Enterobacteriaceae | Initial | 4_days-normal | 0.143159220017511 | 0.0862048983145341 | 0.029 | 1 |
| Enterobacteriaceae | Initial | 10_days-normal | 0.143159220017511 | 0.0531746867133362 | 0.008 | 1 |
| Enterobacteriaceae | 4_days-pepper | 4_days-pepperless | 0.0879871554213148 | 0.0856580124227717 | 0.931 | 1 |
| Enterobacteriaceae | 4_days-pepper | 4_days-normal | 0.0879871554213148 | 0.0862048983145341 | 1 | 1 |
| Enterobacteriaceae | 4_days-pepper | 10_days-normal | 0.0879871554213148 | 0.0531746867133362 | 0.19 | 1 |
| Enterobacteriaceae | 4_days-pepperless | 4_days-normal | 0.0856580124227717 | 0.0862048983145341 | 0.905 | 1 |
| Enterobacteriaceae | 4_days-pepperless | 10_days-normal | 0.0856580124227717 | 0.0531746867133362 | 0.136 | 1 |
| Enterobacteriaceae | 4_days-normal | 10_days-normal | 0.0862048983145341 | 0.0531746867133362 | 0.079 | 1 |
| Enterococcaceae | Initial | 4_days-pepper | 0.000964232091025655 | 0.00028323892035988 | 0.667 | 1 |
| Enterococcaceae | Initial | 4_days-pepperless | 0.000964232091025655 | 0.00141413385404939 | 1 | 1 |
| Enterococcaceae | Initial | 4_days-normal | 0.000964232091025655 | 0.00133906611114328 | 0.267 | 1 |
| Enterococcaceae | Initial | 10_days-normal | 0.000964232091025655 | 0.000508841372243853 | 0.333 | 1 |
| Enterococcaceae | 4_days-pepper | 4_days-pepperless | 0.00028323892035988 | 0.00141413385404939 | 0.5 | 1 |
| Enterococcaceae | 4_days-pepper | 4_days-normal | 0.00028323892035988 | 0.00133906611114328 | 0.8 | 1 |
| Enterococcaceae | 4_days-pepper | 10_days-normal | 0.00028323892035988 | 0.000508841372243853 | 1 | 1 |
| Enterococcaceae | 4_days-pepperless | 4_days-normal | 0.00141413385404939 | 0.00133906611114328 | 0.4 | 1 |
| Enterococcaceae | 4_days-pepperless | 10_days-normal | 0.00141413385404939 | 0.000508841372243853 | 0.4 | 1 |
| Enterococcaceae | 4_days-normal | 10_days-normal | 0.00133906611114328 | 0.000508841372243853 | 0.267 | 1 |
| Erysipelotrichaceae | Initial | 4_days-pepper | 0.00180020309222978 | 0.00323472811339231 | 0.629 | 1 |
| Erysipelotrichaceae | Initial | 4_days-pepperless | 0.00180020309222978 | 0.0156956105790097 | 0.114 | 1 |
| Erysipelotrichaceae | Initial | 4_days-normal | 0.00180020309222978 | 0.00477021000213966 | 0.808 | 1 |
| Erysipelotrichaceae | Initial | 10_days-normal | 0.00180020309222978 | 0.0115986381400899 | 0.683 | 1 |
| Erysipelotrichaceae | 4_days-pepper | 4_days-pepperless | 0.00323472811339231 | 0.0156956105790097 | 0.381 | 1 |
| Erysipelotrichaceae | 4_days-pepper | 4_days-normal | 0.00323472811339231 | 0.00477021000213966 | 0.776 | 1 |
| Erysipelotrichaceae | 4_days-pepper | 10_days-normal | 0.00323472811339231 | 0.0115986381400899 | 1 | 1 |
| Erysipelotrichaceae | 4_days-pepperless | 4_days-normal | 0.0156956105790097 | 0.00477021000213966 | 0.108 | 1 |
| Erysipelotrichaceae | 4_days-pepperless | 10_days-normal | 0.0156956105790097 | 0.0115986381400899 | 0.108 | 1 |
| Erysipelotrichaceae | 4_days-normal | 10_days-normal | 0.00477021000213966 | 0.0115986381400899 | 0.645 | 1 |
| Helicobacteraceae | Initial | 4_days-pepper | 0.00103591575650281 | 0.00186644158888589 | 0.73 | 1 |
| Helicobacteraceae | Initial | 4_days-pepperless | 0.00103591575650281 | 0.000939369644135502 | 0.686 | 1 |
| Helicobacteraceae | Initial | 4_days-normal | 0.00103591575650281 | 0.000791344064874292 | 0.886 | 1 |
| Helicobacteraceae | Initial | 10_days-normal | 0.00103591575650281 | 0.000482301613423838 | 0.064 | 1 |
| Helicobacteraceae | 4_days-pepper | 4_days-pepperless | 0.00186644158888589 | 0.000939369644135502 | 0.73 | 1 |
| Helicobacteraceae | 4_days-pepper | 4_days-normal | 0.00186644158888589 | 0.000791344064874292 | 0.73 | 1 |
| Helicobacteraceae | 4_days-pepper | 10_days-normal | 0.00186644158888589 | 0.000482301613423838 | 0.095 | 1 |
| Helicobacteraceae | 4_days-pepperless | 4_days-normal | 0.000939369644135502 | 0.000791344064874292 | 1 | 1 |
| Helicobacteraceae | 4_days-pepperless | 10_days-normal | 0.000939369644135502 | 0.000482301613423838 | 0.111 | 1 |
| Helicobacteraceae | 4_days-normal | 10_days-normal | 0.000791344064874292 | 0.000482301613423838 | 0.111 | 1 |
| Lachnospiraceae | Initial | 4_days-pepper | 0.0116406089321087 | 0.111820862838048 | 0.083 | 1 |
| Lachnospiraceae | Initial | 4_days-pepperless | 0.0116406089321087 | 0.0487618445358704 | 0.481 | 1 |
| Lachnospiraceae | Initial | 4_days-normal | 0.0116406089321087 | 0.0348102294414086 | 0.315 | 1 |
| Lachnospiraceae | Initial | 10_days-normal | 0.0116406089321087 | 0.0811670100013454 | 0.573 | 1 |
| Lachnospiraceae | 4_days-pepper | 4_days-pepperless | 0.111820862838048 | 0.0487618445358704 | 0.278 | 1 |
| Lachnospiraceae | 4_days-pepper | 4_days-normal | 0.111820862838048 | 0.0348102294414086 | 0.218 | 1 |
| Lachnospiraceae | 4_days-pepper | 10_days-normal | 0.111820862838048 | 0.0811670100013454 | 0.481 | 1 |
| Lachnospiraceae | 4_days-pepperless | 4_days-normal | 0.0487618445358704 | 0.0348102294414086 | 0.968 | 1 |
| Lachnospiraceae | 4_days-pepperless | 10_days-normal | 0.0487618445358704 | 0.0811670100013454 | 0.968 | 1 |
| Lachnospiraceae | 4_days-normal | 10_days-normal | 0.0348102294414086 | 0.0811670100013454 | 0.796 | 1 |
| Lactobacillaceae | Initial | 4_days-pepper | 0.0200901818037032 | 0.0151100608969392 | 0.481 | 1 |
| Lactobacillaceae | Initial | 4_days-pepperless | 0.0200901818037032 | 0.0243201544436315 | 0.236 | 1 |
| Lactobacillaceae | Initial | 4_days-normal | 0.0200901818037032 | 0.040451545438293 | 0.546 | 1 |
| Lactobacillaceae | Initial | 10_days-normal | 0.0200901818037032 | 0.0960523682721246 | 0.863 | 1 |
| Lactobacillaceae | 4_days-pepper | 4_days-pepperless | 0.0151100608969392 | 0.0243201544436315 | 0.105 | 1 |
| Lactobacillaceae | 4_days-pepper | 4_days-normal | 0.0151100608969392 | 0.040451545438293 | 0.2 | 1 |
| Lactobacillaceae | 4_days-pepper | 10_days-normal | 0.0151100608969392 | 0.0960523682721246 | 0.541 | 1 |
| Lactobacillaceae | 4_days-pepperless | 4_days-normal | 0.0243201544436315 | 0.040451545438293 | 0.888 | 1 |
| Lactobacillaceae | 4_days-pepperless | 10_days-normal | 0.0243201544436315 | 0.0960523682721246 | 0.606 | 1 |
| Lactobacillaceae | 4_days-normal | 10_days-normal | 0.040451545438293 | 0.0960523682721246 | 1 | 1 |
| Leuconostocaceae | Initial | 4_days-normal | 2,59E+09 | 8,88E+09 | 1 | 1 |
| Leuconostocaceae | Initial | 10_days-normal | 2,59E+09 | 4,61E+08 | 1 | 1 |
| Leuconostocaceae | 4_days-normal | 10_days-normal | 8,88E+09 | 4,61E+08 | 1 | 1 |
| Methylocystaceae | 4_days-pepper | 4_days-pepperless | 8,33E+09 | 5,90E+09 | 1 | 1 |
| Methylocystaceae | 4_days-pepper | 10_days-normal | 8,33E+09 | 0.000192215909047381 | 1 | 1 |
| Methylocystaceae | 4_days-pepperless | 10_days-normal | 5,90E+09 | 0.000192215909047381 | 1 | 1 |
| Microbacteriaceae | Initial | 4_days-pepper | 0.00732486672748528 | 0.00348604801389244 | 0.035 | 1 |
| Microbacteriaceae | Initial | 4_days-pepperless | 0.00732486672748528 | 0.00574621941283967 | 0.014 | 1 |
| Microbacteriaceae | Initial | 4_days-normal | 0.00732486672748528 | 0.00661190538269945 | 0.408 | 1 |
| Microbacteriaceae | Initial | 10_days-normal | 0.00732486672748528 | 0.00364153685411721 | 0.007 | 1 |
| Microbacteriaceae | 4_days-pepper | 4_days-pepperless | 0.00348604801389244 | 0.00574621941283967 | 0.662 | 1 |
| Microbacteriaceae | 4_days-pepper | 4_days-normal | 0.00348604801389244 | 0.00661190538269945 | 0.328 | 1 |
| Microbacteriaceae | 4_days-pepper | 10_days-normal | 0.00348604801389244 | 0.00364153685411721 | 0.945 | 1 |
| Microbacteriaceae | 4_days-pepperless | 4_days-normal | 0.00574621941283967 | 0.00661190538269945 | 0.423 | 1 |
| Microbacteriaceae | 4_days-pepperless | 10_days-normal | 0.00574621941283967 | 0.00364153685411721 | 0.232 | 1 |
| Microbacteriaceae | 4_days-normal | 10_days-normal | 0.00661190538269945 | 0.00364153685411721 | 0.142 | 1 |
| Micrococcaceae | Initial | 4_days-pepperless | 0.000155212554971113 | 0.000509566910299345 | 1 | 1 |
| Micrococcaceae | Initial | 4_days-normal | 0.000155212554971113 | 0.000767326164933476 | 0.8 | 1 |
| Micrococcaceae | Initial | 10_days-normal | 0.000155212554971113 | 0.000192886125356742 | 0.667 | 1 |
| Micrococcaceae | 4_days-pepperless | 4_days-normal | 0.000509566910299345 | 0.000767326164933476 | 0.629 | 1 |
| Micrococcaceae | 4_days-pepperless | 10_days-normal | 0.000509566910299345 | 0.000192886125356742 | 0.8 | 1 |
| Micrococcaceae | 4_days-normal | 10_days-normal | 0.000767326164933476 | 0.000192886125356742 | 0.267 | 1 |
| Moraxellaceae | Initial | 4_days-pepper | 0.00155427417478159 | 0.000166611129623459 | 0.4 | 1 |
| Moraxellaceae | Initial | 4_days-pepperless | 0.00155427417478159 | 0.00135976945953868 | 0.028 | 1 |
| Moraxellaceae | Initial | 4_days-normal | 0.00155427417478159 | 0.00196550515205051 | 0.914 | 1 |
| Moraxellaceae | Initial | 10_days-normal | 0.00155427417478159 | 0.00027126820632576 | 0.029 | 1 |
| Moraxellaceae | 4_days-pepper | 4_days-pepperless | 0.000166611129623459 | 0.00135976945953868 | 0.889 | 1 |
| Moraxellaceae | 4_days-pepper | 4_days-normal | 0.000166611129623459 | 0.00196550515205051 | 0.571 | 1 |
| Moraxellaceae | 4_days-pepper | 10_days-normal | 0.000166611129623459 | 0.00027126820632576 | 0.8 | 1 |
| Moraxellaceae | 4_days-pepperless | 4_days-normal | 0.00135976945953868 | 0.00196550515205051 | 0.081 | 1 |
| Moraxellaceae | 4_days-pepperless | 10_days-normal | 0.00135976945953868 | 0.00027126820632576 | 0.073 | 1 |
| Moraxellaceae | 4_days-normal | 10_days-normal | 0.00196550515205051 | 0.00027126820632576 | 0.067 | 1 |
| Mycoplasmataceae | Initial | 4_days-pepper | 0.279810801507548 | 0.168408630738045 | 0.024 | 1 |
| Mycoplasmataceae | Initial | 4_days-pepperless | 0.279810801507548 | 0.224889625012347 | 0.19 | 1 |
| Mycoplasmataceae | Initial | 4_days-normal | 0.279810801507548 | 0.23201117072446 | 0.497 | 1 |
| Mycoplasmataceae | Initial | 10_days-normal | 0.279810801507548 | 0.167292996386348 | 0.062 | 1 |
| Mycoplasmataceae | 4_days-pepper | 4_days-pepperless | 0.168408630738045 | 0.224889625012347 | 0.297 | 1 |
| Mycoplasmataceae | 4_days-pepper | 4_days-normal | 0.168408630738045 | 0.23201117072446 | 0.243 | 1 |
| Mycoplasmataceae | 4_days-pepper | 10_days-normal | 0.168408630738045 | 0.167292996386348 | 0.796 | 1 |
| Mycoplasmataceae | 4_days-pepperless | 4_days-normal | 0.224889625012347 | 0.23201117072446 | 0.661 | 1 |
| Mycoplasmataceae | 4_days-pepperless | 10_days-normal | 0.224889625012347 | 0.167292996386348 | 0.436 | 1 |
| Mycoplasmataceae | 4_days-normal | 10_days-normal | 0.23201117072446 | 0.167292996386348 | 0.315 | 1 |
| Peptostreptococcaceae | 4_days-pepperless | 4_days-normal | 0.000788090467749495 | 4,09E+09 | 0.5 | 1 |
| Peptostreptococcaceae | 4_days-pepperless | 10_days-normal | 0.000788090467749495 | 0.00100900114359616 | 1 | 1 |
| Peptostreptococcaceae | 4_days-normal | 10_days-normal | 4,09E+09 | 0.00100900114359616 | 0.667 | 1 |
| Porphyromonadaceae | Initial | 4_days-pepper | 0.00129652372052012 | 0.00667525409385246 | 0.019 | 1 |
| Porphyromonadaceae | Initial | 4_days-pepperless | 0.00129652372052012 | 0.00307508007458649 | 0.257 | 1 |
| Porphyromonadaceae | Initial | 4_days-normal | 0.00129652372052012 | 0.00909943902000573 | 0.683 | 1 |
| Porphyromonadaceae | Initial | 10_days-normal | 0.00129652372052012 | 0.0099920349162816 | 0.927 | 1 |
| Porphyromonadaceae | 4_days-pepper | 4_days-pepperless | 0.00667525409385246 | 0.00307508007458649 | 0.132 | 1 |
| Porphyromonadaceae | 4_days-pepper | 4_days-normal | 0.00667525409385246 | 0.00909943902000573 | 0.043 | 1 |
| Porphyromonadaceae | 4_days-pepper | 10_days-normal | 0.00667525409385246 | 0.0099920349162816 | 0.295 | 1 |
| Porphyromonadaceae | 4_days-pepperless | 4_days-normal | 0.00307508007458649 | 0.00909943902000573 | 0.228 | 1 |
| Porphyromonadaceae | 4_days-pepperless | 10_days-normal | 0.00307508007458649 | 0.0099920349162816 | 0.731 | 1 |
| Porphyromonadaceae | 4_days-normal | 10_days-normal | 0.00909943902000573 | 0.0099920349162816 | 0.955 | 1 |
| Prevotellaceae | 4_days-pepper | 4_days-pepperless | 0.0107002815443005 | 0.00268551006004017 | 0.286 | 1 |
| Prevotellaceae | 4_days-pepper | 4_days-normal | 0.0107002815443005 | 7,40E+08 | 0.133 | 1 |
| Prevotellaceae | 4_days-pepper | 10_days-normal | 0.0107002815443005 | 0.000212539851222104 | 0.8 | 1 |
| Prevotellaceae | 4_days-pepperless | 4_days-normal | 0.00268551006004017 | 7,40E+08 | 0.095 | 1 |
| Prevotellaceae | 4_days-pepperless | 10_days-normal | 0.00268551006004017 | 0.000212539851222104 | 0.667 | 1 |
| Prevotellaceae | 4_days-normal | 10_days-normal | 7,40E+08 | 0.000212539851222104 | 0.667 | 1 |
| Pseudomonadaceae | Initial | 4_days-pepperless | 0.0021378477623494 | 0.00262152035778345 | 0.352 | 1 |
| Pseudomonadaceae | Initial | 4_days-normal | 0.0021378477623494 | 0.00180650010090339 | 0.686 | 1 |
| Pseudomonadaceae | Initial | 10_days-normal | 0.0021378477623494 | 0.00121964507289134 | 0.114 | 1 |
| Pseudomonadaceae | 4_days-pepperless | 4_days-normal | 0.00262152035778345 | 0.00180650010090339 | 0.61 | 1 |
| Pseudomonadaceae | 4_days-pepperless | 10_days-normal | 0.00262152035778345 | 0.00121964507289134 | 0.132 | 1 |
| Pseudomonadaceae | 4_days-normal | 10_days-normal | 0.00180650010090339 | 0.00121964507289134 | 0.114 | 1 |
| Rhabdochlamydiaceae | Initial | 4_days-pepperless | 0.000428125044404144 | 0.001609051448132 | 0.381 | 1 |
| Rhabdochlamydiaceae | Initial | 4_days-normal | 0.000428125044404144 | 0.00118648598832007 | 1 | 1 |
| Rhabdochlamydiaceae | Initial | 10_days-normal | 0.000428125044404144 | 0.000558115640212541 | 0.381 | 1 |
| Rhabdochlamydiaceae | 4_days-pepperless | 4_days-normal | 0.001609051448132 | 0.00118648598832007 | 0.177 | 1 |
| Rhabdochlamydiaceae | 4_days-pepperless | 10_days-normal | 0.001609051448132 | 0.000558115640212541 | 0.008 | 1 |
| Rhabdochlamydiaceae | 4_days-normal | 10_days-normal | 0.00118648598832007 | 0.000558115640212541 | 0.177 | 1 |
| Rhodobacteraceae | 4_days-pepper | 4_days-pepperless | 0.000228310502283105 | 0.000127459683605997 | 0.667 | 1 |
| Rhodobacteraceae | 4_days-pepper | 4_days-normal | 0.000228310502283105 | 8,21E+09 | 1 | 1 |
| Rhodobacteraceae | 4_days-pepper | 10_days-normal | 0.000228310502283105 | 0.000194485280361903 | 0.5 | 1 |
| Rhodobacteraceae | 4_days-pepperless | 4_days-normal | 0.000127459683605997 | 8,21E+09 | 0.667 | 1 |
| Rhodobacteraceae | 4_days-pepperless | 10_days-normal | 0.000127459683605997 | 0.000194485280361903 | 1 | 1 |
| Rhodobacteraceae | 4_days-normal | 10_days-normal | 8,21E+09 | 0.000194485280361903 | 0.5 | 1 |
| Rikenellaceae | Initial | 4_days-pepper | 0.00208907357719072 | 0.0107642409604635 | 0.247 | 1 |
| Rikenellaceae | Initial | 4_days-pepperless | 0.00208907357719072 | 0.010344680080223 | 0.354 | 1 |
| Rikenellaceae | Initial | 4_days-normal | 0.00208907357719072 | 0.00317831567627469 | 0.639 | 1 |
| Rikenellaceae | Initial | 10_days-normal | 0.00208907357719072 | 0.00885362511240758 | 0.329 | 1 |
| Rikenellaceae | 4_days-pepper | 4_days-pepperless | 0.0107642409604635 | 0.010344680080223 | 0.852 | 1 |
| Rikenellaceae | 4_days-pepper | 4_days-normal | 0.0107642409604635 | 0.00317831567627469 | 0.366 | 1 |
| Rikenellaceae | 4_days-pepper | 10_days-normal | 0.0107642409604635 | 0.00885362511240758 | 0.699 | 1 |
| Rikenellaceae | 4_days-pepperless | 4_days-normal | 0.010344680080223 | 0.00317831567627469 | 0.336 | 1 |
| Rikenellaceae | 4_days-pepperless | 10_days-normal | 0.010344680080223 | 0.00885362511240758 | 1 | 1 |
| Rikenellaceae | 4_days-normal | 10_days-normal | 0.00317831567627469 | 0.00885362511240758 | 0.534 | 1 |
| Ruminococcaceae | Initial | 4_days-pepper | 0.00117397472871068 | 0.00318461482940309 | 0.133 | 1 |
| Ruminococcaceae | Initial | 4_days-pepperless | 0.00117397472871068 | 0.00619830159341987 | 0.788 | 1 |
| Ruminococcaceae | Initial | 4_days-normal | 0.00117397472871068 | 0.0709066021594567 | 0.527 | 1 |
| Ruminococcaceae | Initial | 10_days-normal | 0.00117397472871068 | 0.00782018711497178 | 0.57 | 1 |
| Ruminococcaceae | 4_days-pepper | 4_days-pepperless | 0.00318461482940309 | 0.00619830159341987 | 0.5 | 1 |
| Ruminococcaceae | 4_days-pepper | 4_days-normal | 0.00318461482940309 | 0.0709066021594567 | 0.5 | 1 |
| Ruminococcaceae | 4_days-pepper | 10_days-normal | 0.00318461482940309 | 0.00782018711497178 | 0.267 | 1 |
| Ruminococcaceae | 4_days-pepperless | 4_days-normal | 0.00619830159341987 | 0.0709066021594567 | 0.805 | 1 |
| Ruminococcaceae | 4_days-pepperless | 10_days-normal | 0.00619830159341987 | 0.00782018711497178 | 0.613 | 1 |
| Ruminococcaceae | 4_days-normal | 10_days-normal | 0.0709066021594567 | 0.00782018711497178 | 0.397 | 1 |
| S24-7 | Initial | 4_days-pepper | 0.032196170476555 | 0.0380821718503253 | 0.382 | 1 |
| S24-7 | Initial | 4_days-pepperless | 0.032196170476555 | 0.0594237323238326 | 0.065 | 1 |
| S24-7 | Initial | 4_days-normal | 0.032196170476555 | 0.0299710071870272 | 0.878 | 1 |
| S24-7 | Initial | 10_days-normal | 0.032196170476555 | 0.0194850721949602 | 0.027 | 1 |
| S24-7 | 4_days-pepper | 4_days-pepperless | 0.0380821718503253 | 0.0594237323238326 | 0.195 | 1 |
| S24-7 | 4_days-pepper | 4_days-normal | 0.0380821718503253 | 0.0299710071870272 | 0.328 | 1 |
| S24-7 | 4_days-pepper | 10_days-normal | 0.0380821718503253 | 0.0194850721949602 | 0.2 | 1 |
| S24-7 | 4_days-pepperless | 4_days-normal | 0.0594237323238326 | 0.0299710071870272 | 0.105 | 1 |
| S24-7 | 4_days-pepperless | 10_days-normal | 0.0594237323238326 | 0.0194850721949602 | 0.006 | 1 |
| S24-7 | 4_days-normal | 10_days-normal | 0.0299710071870272 | 0.0194850721949602 | 0.046 | 1 |
| Sphingobacteriaceae | 4_days-pepper | 4_days-normal | 0.000125881168177241 | 1,93E+09 | 1 | 1 |
| Sphingobacteriaceae | 4_days-pepper | 10_days-normal | 0.000125881168177241 | 4,91E+08 | 1 | 1 |
| Sphingobacteriaceae | 4_days-normal | 10_days-normal | 1,93E+09 | 4,91E+08 | 1 | 1 |
| Staphylococcaceae | Initial | 4_days-pepper | 0.0487944869592154 | 0.0255023317338127 | 0.027 | 1 |
| Staphylococcaceae | Initial | 4_days-pepperless | 0.0487944869592154 | 0.0237512998565907 | 0.001 | 0.417 |
| Staphylococcaceae | Initial | 4_days-normal | 0.0487944869592154 | 0.0322893126885634 | 0.022 | 1 |
| Staphylococcaceae | Initial | 10_days-normal | 0.0487944869592154 | 0.0127481147134046 | 0.000699 | 0.292182 |
| Staphylococcaceae | 4_days-pepper | 4_days-pepperless | 0.0255023317338127 | 0.0237512998565907 | 0.673 | 1 |
| Staphylococcaceae | 4_days-pepper | 4_days-normal | 0.0255023317338127 | 0.0322893126885634 | 1 | 1 |
| Staphylococcaceae | 4_days-pepper | 10_days-normal | 0.0255023317338127 | 0.0127481147134046 | 0.152 | 1 |
| Staphylococcaceae | 4_days-pepperless | 4_days-normal | 0.0237512998565907 | 0.0322893126885634 | 0.356 | 1 |
| Staphylococcaceae | 4_days-pepperless | 10_days-normal | 0.0237512998565907 | 0.0127481147134046 | 0.299 | 1 |
| Staphylococcaceae | 4_days-normal | 10_days-normal | 0.0322893126885634 | 0.0127481147134046 | 0.161 | 1 |
| Streptococcaceae | Initial | 4_days-pepper | 0.00107888573107295 | 0.00145541029794365 | 1 | 1 |
| Streptococcaceae | Initial | 4_days-pepperless | 0.00107888573107295 | 0.00345537786285378 | 0.786 | 1 |
| Streptococcaceae | Initial | 4_days-normal | 0.00107888573107295 | 0.00124818497100346 | 1 | 1 |
| Streptococcaceae | Initial | 10_days-normal | 0.00107888573107295 | 0.00888339955489009 | 1 | 1 |
| Streptococcaceae | 4_days-pepper | 4_days-pepperless | 0.00145541029794365 | 0.00345537786285378 | 0.857 | 1 |
| Streptococcaceae | 4_days-pepper | 4_days-normal | 0.00145541029794365 | 0.00124818497100346 | 1 | 1 |
| Streptococcaceae | 4_days-pepper | 10_days-normal | 0.00145541029794365 | 0.00888339955489009 | 1 | 1 |
| Streptococcaceae | 4_days-pepperless | 4_days-normal | 0.00345537786285378 | 0.00124818497100346 | 0.421 | 1 |
| Streptococcaceae | 4_days-pepperless | 10_days-normal | 0.00345537786285378 | 0.00888339955489009 | 0.662 | 1 |
| Streptococcaceae | 4_days-normal | 10_days-normal | 0.00124818497100346 | 0.00888339955489009 | 1 | 1 |
| Thermaceae | Initial | 4_days-pepperless | 0.000250889090575562 | 0.000526752618446933 | 1 | 1 |
| Thermaceae | Initial | 4_days-normal | 0.000250889090575562 | 0.000592961268284589 | 0.533 | 1 |
| Thermaceae | Initial | 10_days-normal | 0.000250889090575562 | 0.000683778655785217 | 0.8 | 1 |
| Thermaceae | 4_days-pepperless | 4_days-normal | 0.000526752618446933 | 0.000592961268284589 | 0.343 | 1 |
| Thermaceae | 4_days-pepperless | 10_days-normal | 0.000526752618446933 | 0.000683778655785217 | 0.886 | 1 |
| Thermaceae | 4_days-normal | 10_days-normal | 0.000592961268284589 | 0.000683778655785217 | 0.686 | 1 |
| Turicibacteraceae | 4_days-pepperless | 4_days-normal | 0.00550983773946851 | 0.00124237960035785 | 0.111 | 1 |
| Turicibacteraceae | 4_days-pepperless | 10_days-normal | 0.00550983773946851 | 1,50E+09 | 0.333 | 1 |
| Turicibacteraceae | 4_days-normal | 10_days-normal | 0.00124237960035785 | 1,50E+09 | 0.4 | 1 |
| Veillonellaceae | Initial | 4_days-pepper | 0.000826536044617065 | 0.00142095939798054 | 0.4 | 1 |
| Veillonellaceae | Initial | 4_days-normal | 0.000826536044617065 | 0.000143059323635955 | 1 | 1 |
| Veillonellaceae | 4_days-pepper | 4_days-normal | 0.00142095939798054 | 0.000143059323635955 | 0.667 | 1 |
| Verrucomicrobiaceae | Initial | 4_days-pepper | 0.0197130442927656 | 0.0593842425680258 | 0.73 | 1 |
| Verrucomicrobiaceae | Initial | 4_days-pepperless | 0.0197130442927656 | 0.0160911297092805 | 0.546 | 1 |
| Verrucomicrobiaceae | Initial | 4_days-normal | 0.0197130442927656 | 0.014404740350864 | 0.489 | 1 |
| Verrucomicrobiaceae | Initial | 10_days-normal | 0.0197130442927656 | 0.0073239152444605 | 0.015 | 1 |
| Verrucomicrobiaceae | 4_days-pepper | 4_days-pepperless | 0.0593842425680258 | 0.0160911297092805 | 0.436 | 1 |
| Verrucomicrobiaceae | 4_days-pepper | 4_days-normal | 0.0593842425680258 | 0.014404740350864 | 0.436 | 1 |
| Verrucomicrobiaceae | 4_days-pepper | 10_days-normal | 0.0593842425680258 | 0.0073239152444605 | 0.027 | 1 |
| Verrucomicrobiaceae | 4_days-pepperless | 4_days-normal | 0.0160911297092805 | 0.014404740350864 | 0.666 | 1 |
| Verrucomicrobiaceae | 4_days-pepperless | 10_days-normal | 0.0160911297092805 | 0.0073239152444605 | 0.027 | 1 |
| Verrucomicrobiaceae | 4_days-normal | 10_days-normal | 0.014404740350864 | 0.0073239152444605 | 0.074 | 1 |
| Williamsiaceae | Initial | 4_days-pepperless | 0.000247923181926728 | 0.000196506405347812 | 0.667 | 1 |
| Williamsiaceae | Initial | 4_days-normal | 0.000247923181926728 | 0.00039962369348928 | 1 | 1 |
| Williamsiaceae | Initial | 10_days-normal | 0.000247923181926728 | 0.000165367368807251 | 0.4 | 1 |
| Williamsiaceae | 4_days-pepperless | 4_days-normal | 0.000196506405347812 | 0.00039962369348928 | 1 | 1 |
| Williamsiaceae | 4_days-pepperless | 10_days-normal | 0.000196506405347812 | 0.000165367368807251 | 0.8 | 1 |
| Williamsiaceae | 4_days-normal | 10_days-normal | 0.00039962369348928 | 0.000165367368807251 | 0.4 | 1 |
| Xanthomonadaceae | Initial | 4_days-pepperless | 0.000156587387550309 | 7,89E+08 | 1 | 1 |
| Xanthomonadaceae | Initial | 4_days-normal | 0.000156587387550309 | 5,62E+09 | 0.667 | 1 |
| Xanthomonadaceae | Initial | 10_days-normal | 0.000156587387550309 | 5,38E+09 | 0.667 | 1 |
| Xanthomonadaceae | 4_days-pepperless | 4_days-normal | 7,89E+08 | 5,62E+09 | 1 | 1 |
| Xanthomonadaceae | 4_days-pepperless | 10_days-normal | 7,89E+08 | 5,38E+09 | 1 | 1 |
| Xanthomonadaceae | 4_days-normal | 10_days-normal | 5,62E+09 | 5,38E+09 | 1 | 1 |
| [Mogibacteriaceae] | 4_days-pepperless | 4_days-normal | 0.000638649266711966 | 0.000138721908813465 | 1 | 1 |
| [Odoribacteraceae] | Initial | 4_days-pepper | 0.000799629157492177 | 0.00509875092830262 | 0.667 | 1 |
| [Odoribacteraceae] | Initial | 4_days-pepperless | 0.000799629157492177 | 0.000133608648123406 | 1 | 1 |
| [Odoribacteraceae] | Initial | 4_days-normal | 0.000799629157492177 | 0.0107751001807717 | 1 | 1 |
| [Odoribacteraceae] | Initial | 10_days-normal | 0.000799629157492177 | 0.00746093214459516 | 1 | 1 |
| [Odoribacteraceae] | 4_days-pepper | 4_days-pepperless | 0.00509875092830262 | 0.000133608648123406 | 0.667 | 1 |
| [Odoribacteraceae] | 4_days-pepper | 4_days-normal | 0.00509875092830262 | 0.0107751001807717 | 1 | 1 |
| [Odoribacteraceae] | 4_days-pepper | 10_days-normal | 0.00509875092830262 | 0.00746093214459516 | 0.222 | 1 |
| [Odoribacteraceae] | 4_days-pepperless | 4_days-normal | 0.000133608648123406 | 0.0107751001807717 | 0.8 | 1 |
| [Odoribacteraceae] | 4_days-pepperless | 10_days-normal | 0.000133608648123406 | 0.00746093214459516 | 0.5 | 1 |
| [Odoribacteraceae] | 4_days-normal | 10_days-normal | 0.0107751001807717 | 0.00746093214459516 | 0.788 | 1 |
