# Supplementary material for: Pepper power: short-term impact of pepper consumption on the gut bacteriome composition in healthy volunteers
Source: PeerJ. 2024 Dec 13;12:e18707. doi: 10.7717/peerj.18707 (PMC11648697; doi:10.7717/peerj.18707)
Supplement: Supplemental Information 3 [file peerj-12-18707-s003.docx]

| Phylum | group1 | group2 | AbundanceGroup1 | AbundanceGroup2 | p | p.adj |
| --- | --- | --- | --- | --- | --- | --- |
| Actinobacteria | Initial | 4_days-pepper | 0.229316729602451 | 0.137827747081476 | 0.054 | 1 |
| Actinobacteria | Initial | 4_days-pepperless | 0.229316729602451 | 0.19942237330034 | 0.278 | 1 |
| Actinobacteria | Initial | 4_days-normal | 0.229316729602451 | 0.193556717566207 | 0.447 | 1 |
| Actinobacteria | Initial | 10_days-normal | 0.229316729602451 | 0.14228247970544 | 0.077 | 1 |
| Actinobacteria | 4_days-pepper | 4_days-pepperless | 0.137827747081476 | 0.19942237330034 | 0.28 | 1 |
| Actinobacteria | 4_days-pepper | 4_days-normal | 0.137827747081476 | 0.193556717566207 | 0.218 | 1 |
| Actinobacteria | 4_days-pepper | 10_days-normal | 0.137827747081476 | 0.14228247970544 | 0.842 | 1 |
| Actinobacteria | 4_days-pepperless | 4_days-normal | 0.19942237330034 | 0.193556717566207 | 0.796 | 1 |
| Actinobacteria | 4_days-pepperless | 10_days-normal | 0.19942237330034 | 0.14228247970544 | 0.447 | 1 |
| Actinobacteria | 4_days-normal | 10_days-normal | 0.193556717566207 | 0.14228247970544 | 0.278 | 1 |
| Bacteroidetes | Initial | 4_days-pepper | 0.214342731427416 | 0.353747083412177 | 0.123 | 1 |
| Bacteroidetes | Initial | 4_days-pepperless | 0.214342731427416 | 0.300832829315265 | 0.123 | 1 |
| Bacteroidetes | Initial | 4_days-normal | 0.214342731427416 | 0.182586604573765 | 0.739 | 1 |
| Bacteroidetes | Initial | 10_days-normal | 0.214342731427416 | 0.332374818670638 | 0.315 | 1 |
| Bacteroidetes | 4_days-pepper | 4_days-pepperless | 0.353747083412177 | 0.300832829315265 | 0.529 | 1 |
| Bacteroidetes | 4_days-pepper | 4_days-normal | 0.353747083412177 | 0.182586604573765 | 0.052 | 1 |
| Bacteroidetes | 4_days-pepper | 10_days-normal | 0.353747083412177 | 0.332374818670638 | 0.796 | 1 |
| Bacteroidetes | 4_days-pepperless | 4_days-normal | 0.300832829315265 | 0.182586604573765 | 0.063 | 1 |
| Bacteroidetes | 4_days-pepperless | 10_days-normal | 0.300832829315265 | 0.332374818670638 | 0.796 | 1 |
| Bacteroidetes | 4_days-normal | 10_days-normal | 0.182586604573765 | 0.332374818670638 | 0.19 | 1 |
| Chlamydiae | Initial | 4_days-pepperless | 0.000420520385554137 | 0.00156393233155317 | 0.571 | 1 |
| Chlamydiae | Initial | 4_days-normal | 0.000420520385554137 | 0.00112850447718294 | 1 | 1 |
| Chlamydiae | Initial | 10_days-normal | 0.000420520385554137 | 0.000505833952241666 | 0.381 | 1 |
| Chlamydiae | 4_days-pepperless | 4_days-normal | 0.00156393233155317 | 0.00112850447718294 | 0.177 | 1 |
| Chlamydiae | 4_days-pepperless | 10_days-normal | 0.00156393233155317 | 0.000505833952241666 | 0.008 | 0.912 |
| Chlamydiae | 4_days-normal | 10_days-normal | 0.00112850447718294 | 0.000505833952241666 | 0.247 | 1 |
| Firmicutes | Initial | 4_days-pepper | 0.0949411436655867 | 0.188341032506045 | 0.393 | 1 |
| Firmicutes | Initial | 4_days-pepperless | 0.0949411436655867 | 0.151467400482046 | 0.447 | 1 |
| Firmicutes | Initial | 4_days-normal | 0.0949411436655867 | 0.260147848104586 | 0.000788 | 0.09062 |
| Firmicutes | Initial | 10_days-normal | 0.0949411436655867 | 0.293524173845522 | 0.105 | 1 |
| Firmicutes | 4_days-pepper | 4_days-pepperless | 0.188341032506045 | 0.151467400482046 | 0.968 | 1 |
| Firmicutes | 4_days-pepper | 4_days-normal | 0.188341032506045 | 0.260147848104586 | 0.173 | 1 |
| Firmicutes | 4_days-pepper | 10_days-normal | 0.188341032506045 | 0.293524173845522 | 0.436 | 1 |
| Firmicutes | 4_days-pepperless | 4_days-normal | 0.151467400482046 | 0.260147848104586 | 0.37 | 1 |
| Firmicutes | 4_days-pepperless | 10_days-normal | 0.151467400482046 | 0.293524173845522 | 0.447 | 1 |
| Firmicutes | 4_days-normal | 10_days-normal | 0.260147848104586 | 0.293524173845522 | 0.973 | 1 |
| Proteobacteria | Initial | 4_days-pepper | 0.157540508445286 | 0.100541752930266 | 0.075 | 1 |
| Proteobacteria | Initial | 4_days-pepperless | 0.157540508445286 | 0.107397733338308 | 0.133 | 1 |
| Proteobacteria | Initial | 4_days-normal | 0.157540508445286 | 0.0999435687287784 | 0.063 | 1 |
| Proteobacteria | Initial | 10_days-normal | 0.157540508445286 | 0.0630698994195738 | 0.013 | 1 |
| Proteobacteria | 4_days-pepper | 4_days-pepperless | 0.100541752930266 | 0.107397733338308 | 0.497 | 1 |
| Proteobacteria | 4_days-pepper | 4_days-normal | 0.100541752930266 | 0.0999435687287784 | 0.739 | 1 |
| Proteobacteria | 4_days-pepper | 10_days-normal | 0.100541752930266 | 0.0630698994195738 | 0.315 | 1 |
| Proteobacteria | 4_days-pepperless | 4_days-normal | 0.107397733338308 | 0.0999435687287784 | 0.968 | 1 |
| Proteobacteria | 4_days-pepperless | 10_days-normal | 0.107397733338308 | 0.0630698994195738 | 0.05 | 1 |
| Proteobacteria | 4_days-normal | 10_days-normal | 0.0999435687287784 | 0.0630698994195738 | 0.133 | 1 |
| Tenericutes | Initial | 4_days-pepper | 0.276967008980925 | 0.162055762934576 | 0.024 | 1 |
| Tenericutes | Initial | 4_days-pepperless | 0.276967008980925 | 0.219515445512393 | 0.094 | 1 |
| Tenericutes | Initial | 4_days-normal | 0.276967008980925 | 0.22221058009646 | 0.356 | 1 |
| Tenericutes | Initial | 10_days-normal | 0.276967008980925 | 0.158435574761823 | 0.05 | 1 |
| Tenericutes | 4_days-pepper | 4_days-pepperless | 0.162055762934576 | 0.219515445512393 | 0.297 | 1 |
| Tenericutes | 4_days-pepper | 4_days-normal | 0.162055762934576 | 0.22221058009646 | 0.497 | 1 |
| Tenericutes | 4_days-pepper | 10_days-normal | 0.162055762934576 | 0.158435574761823 | 0.931 | 1 |
| Tenericutes | 4_days-pepperless | 4_days-normal | 0.219515445512393 | 0.22221058009646 | 0.78 | 1 |
| Tenericutes | 4_days-pepperless | 10_days-normal | 0.219515445512393 | 0.158435574761823 | 0.297 | 1 |
| Tenericutes | 4_days-normal | 10_days-normal | 0.22221058009646 | 0.158435574761823 | 0.315 | 1 |
| Verrucomicrobia | Initial | 4_days-pepper | 0.019438760379152 | 0.0563518752212016 | 0.73 | 1 |
| Verrucomicrobia | Initial | 4_days-pepperless | 0.019438760379152 | 0.015620462837804 | 0.489 | 1 |
| Verrucomicrobia | Initial | 4_days-normal | 0.019438760379152 | 0.013400594312991 | 0.489 | 1 |
| Verrucomicrobia | Initial | 10_days-normal | 0.019438760379152 | 0.00686581052615177 | 0.015 | 1 |
| Verrucomicrobia | 4_days-pepper | 4_days-pepperless | 0.0563518752212016 | 0.015620462837804 | 0.34 | 1 |
| Verrucomicrobia | 4_days-pepper | 4_days-normal | 0.0563518752212016 | 0.013400594312991 | 0.436 | 1 |
| Verrucomicrobia | 4_days-pepper | 10_days-normal | 0.0563518752212016 | 0.00686581052615177 | 0.027 | 1 |
| Verrucomicrobia | 4_days-pepperless | 4_days-normal | 0.015620462837804 | 0.013400594312991 | 0.73 | 1 |
| Verrucomicrobia | 4_days-pepperless | 10_days-normal | 0.015620462837804 | 0.00686581052615177 | 0.046 | 1 |
| Verrucomicrobia | 4_days-normal | 10_days-normal | 0.013400594312991 | 0.00686581052615177 | 0.093 | 1 |
| [Thermi] | Initial | 4_days-pepperless | 0.000245469053732819 | 0.000497732291722749 | 1 | 1 |
| [Thermi] | Initial | 4_days-normal | 0.000245469053732819 | 0.000561127695306943 | 0.533 | 1 |
| [Thermi] | Initial | 10_days-normal | 0.000245469053732819 | 0.000647868398311979 | 1 | 1 |
| [Thermi] | 4_days-pepperless | 4_days-normal | 0.000497732291722749 | 0.000561127695306943 | 0.343 | 1 |
| [Thermi] | 4_days-pepperless | 10_days-normal | 0.000497732291722749 | 0.000647868398311979 | 0.886 | 1 |
| [Thermi] | 4_days-normal | 10_days-normal | 0.000561127695306943 | 0.000647868398311979 | 0.886 | 1 |
